# Risk factors for traffic violations and serious crash casualties in rural areas: a comparison of urban–rural differences in China

**DOI:** 10.3389/fpubh.2026.1754725

**Published:** 2026-01-29

**Authors:** Guangnan Zhang, Junjie Lin, Baitong Li

**Affiliations:** Center for Studies of Hong Kong, Macao and Pearl River Delta, Institute of Guangdong, Hong Kong and Macao Development Studies, Sun Yat-sen University, Guangzhou, Guangdong, China

**Keywords:** at-fault crash, crash casualties, risk factor, rural area, urban–rural difference

## Abstract

**Introduction:**

Rural areas are typically characterized by poor traffic conditions, less road supervision, and more illegal driving behaviors. These factors lead to significantly higher mortality and increased rates of road traffic crashes in rural areas than in urban areas. Therefore, an in-depth study of traffic risk factors in the rural areas of developing countries with large rural populations and extensive rural road coverage is particularly urgent and important.

**Method:**

Based on the report data of 38,458 traffic crash occurring in rural areas of Guangdong Province between 2006 and 2014 in the Road Traffic Crash Database of the Ministry of Public Security of China, the logit model was used to evaluate the impact of four factors—drivers, vehicles, roads, and environment—on at-fault crash behavior and serious casualties in rural areas. Furthermore, the risk factors for rural and urban areas were comparatively analyzed.

**Results:**

In rural areas, male drivers, drivers without valid licenses, drivers of unsafe vehicles and drivers of trucks are more likely to cause at-fault crashes and cause serious casualties, as are drivers impacted by certain conditions, including sand and gravel roads, mountainous terrain, and nighttime without street lighting. In urban areas, crashes involving sand and gravel roads and single-vehicle crashes have lower casualties, but migrant workers and self-employed individuals are more likely to die or be seriously injured in urban road crashes.

**Conclusion:**

To reduce the impact of road traffic risk factors on at-fault crash behaviors and serious casualties in the rural areas of developing countries, targeted measures should be implemented for drivers, vehicles, and roads. These measures may include focusing on illegal drivers, trucks, and unsafe vehicles for enhanced supervision, as well as prioritizing the construction and improvement in transportation infrastructure such as roads, lighting, and safety mechanisms in rural areas.

## Highlights

- In rural traffic accidents, one-third of drivers lack licenses, and 7.7% of vehicles are in unfit safety status that significantly increase the likelihood of causing accidents and resulting in serious casualties.
- In rural areas, drivers with rural hukou are more likely to cause serious casualties.
- While migrant workers are more likely to cause accidents in rural areas, they are more likely to cause serious casualties in urban areas.
- Compared to urban areas, drivers in rural areas are less likely to engage in risk-compensating behavior on gravel roads, as gravel roads significantly increase the likelihood of accidents and serious casualties in rural areas.

## 1 Introduction

Currently, 45% of the world’s population still lives in rural areas (1). Due to unfavorable road characteristics (2, 3), environmental conditions (4, 5) and more illegal driving behaviors (6), motor vehicle crashes in rural areas compared to urban areas have a greater risk of death (7, 8). Road traffic infrastructure conditions, supervision intensity, and emergency medical capabilities in rural areas in developing countries are inferior to those in developed countries. In addition, China has a larger rural population and longer rural roads than other developing countries. Therefore, given the limited empirical research on the risk factors for road traffic crashes in rural China, an analysis of the risk factors influencing drivers’ at-fault crash behavior and crash severity in rural China is needed.

Compared with rural roads in developed countries, those in China are longer and have wider coverage (9). In addition, compared with urban roads in China, rural roads in China are characterized by issues such as a lack of safety mechanisms (2), inadequate road maintenance (10), and a lack of safety supervision (6), resulting in a significantly higher growth rate of traffic crash mortality (6). Especially in the context of integrated urban and rural transportation construction in China, the significant differences in transportation infrastructure and safety supervision between urban and rural areas (11) further aggravate traffic risks in rural China. Therefore, it is particularly urgent and important to conduct in-depth analysis of road traffic safety issues in the rural areas of China (12). At present, China’s rural population is approximately 509.79 million (13) and includes the vast majority of its low-income groups.^1^ However, inadequate rural transportation networks and road risks further impede the economic and social development of rural China.^2^ According to statistics, dangerous sections impact more than 40% of rural roads; nearly 35% of serious and extremely serious road traffic crashes occur on rural roads.^3^ In 2019, there was a significant difference in the mortality rate of motor vehicle traffic crashes between urban and rural residents. The rate was 10.61 per 100,000 people for urban residents and 16.35 per 100,000 people for rural residents. Among rural residents, the rate for males was as high as 23.74 per 100,000 people, more than double that of urban residents (14). For example, in China, from 2005 to 2010, the fatality rate for crashes in rural areas increased by 106%, while the increase in urban areas was only 4% (6).

This study aims to reduce the incidence and severity of traffic accidents in rural areas of developing countries. Based on the hypothesis that urban–rural dual structures lead to differing traffic risk factors between urban and rural areas, it investigates the risk factors influencing traffic violations and severe fatal accidents in rural Guangdong Province, China, as well as the nature and extent of their impact. The logit model is used to carry out empirical research based on 38,458 traffic crash reports related to rural areas from the Road Traffic Crash Database of the Ministry of Public Security of China from 2006 to 2014. The risk of drivers causing at-fault crashes and severe casualties in rural traffic crashes in China is analyzed from various aspects, including driver factors, vehicle factors, rural road factors, and environmental factors in rural areas. Furthermore, the similarities and differences in road traffic risk factors between rural and urban areas are compared, and feasible suggestions are provided for improving traffic safety in rural areas with high crash risks and poor medical conditions in the context of urban–rural transportation integration.

The rest of this article is structured as follows. Section 2 is a literature review, which evaluates the relevant studies on traffic crashes in rural areas and proposes improvements to address the existing problems. In Section 3, data descriptions and descriptive statistics are presented. Then, an empirical analysis is conducted in Section 4 on the factors influencing driver’s at-fault crash behaviors and serious casualties in rural areas. Section 5 presents discussion and draws conclusions, followed by policy recommendations and prospects for future research directions in Section 6.

## 2 Literature review

To date, the relevant literature has analyzed the factors influencing traffic crashes and casualties in rural areas from four aspects: driver factors in rural areas, characteristics of common vehicles on rural roads, rural road factors, and the driving environment in rural areas.

Drivers in rural areas are more likely to exhibit risky behaviors (15). In particular, illegal behaviors such as speeding and drunk driving are the main factors affecting the severity of traffic crashes in rural areas (16). Individual risk factors in rural areas have been analyzed in the literature mainly from three aspects: demographic characteristics, risk behaviors, and adverse conditions. In terms of demographic characteristics, male drivers in rural areas (8, 17) and young drivers (18) are more likely to be involved in serious road crash injuries, and the severity of crashes is higher in rural areas than in urban areas (19). Drivers’ risk behaviors on rural roads mainly manifest as more speeding and fatigue driving due to insufficient supervision (6, 20) and improper passing due to a lack of road barriers (21). The consequences of crashes are more serious in rural areas than those in urban areas (7, 20). In developed countries, drunk driving and drug driving are important risk factors contributing to the increase in traffic violations and crashes in rural areas (22), with a greater impact on the severity of traffic crashes in urban areas (23). However, traffic crashes in China mainly involve alcohol; furthermore, these crashes are more severe in rural areas (18, 24).

The type of vehicle has a significant impact on the severity of driver injury in rural road crashes (25); trucks (26) and motorcycles (27) have the greatest impacts. Furthermore, not only do trucks and motorcycles increase the likelihood of drivers being injured or killed in rural road crashes, but the severity of crashes involving trucks (28) and motorcycles (29) at faults in rural areas is greater than that in urban areas. Trucks are not suitable for driving in rural areas where there are more curves and slopes (30), as their large size and weight make them more likely to cause crashes with serious casualties (18). Due to their unpredictable driving trajectories (25) and more dangerous driving behaviors (31), motorcyclists are more likely to be sustain serious injuries or fatalities during crashes in rural areas (27).

Roads in rural areas are characterized by many curves and slopes (3), poor pavement conditions (32), a lack of road traffic safety mechanisms (2), and insufficient maintenance capacity (10). Research on road factors for crashes mainly involves three aspects: road alignment and intersections, pavement and road surface conditions, and traffic mechanisms. The existence of curves and slopes significantly increases the likelihood of traffic crashes in rural areas (33, 34). Daytime curve crashes are more likely to occur in urban areas (30), while intersections significantly increase the severity of crashes in rural areas (25). In rural areas, better pavement conditions are associated with a lower likelihood of crash. However, poor pavement conditions can prompt drivers to drive more cautiously (35). Therefore, roads with better asphalt pavement may instead be more likely to cause serious crash injuries (24); furthermore, more cautious driving also makes driving on wet road surfaces in rural areas less likely to cause severe crashes than driving on dry road surfaces (36) and in urban areas (37). In developed countries, the complex traffic conditions (36) and driver distraction (5) at intersections with signal lights increase the probability of crashes in rural areas. However, in developing countries, the issue of missing traffic signals in rural areas needs to be addressed (9); rumble strips can be installed as median strips to reduce the risk of crashes (38).

In terms of temporal factors, the period from 12:00 a.m. and 6:00 a.m., weekdays, and winter conditions are more likely to cause serious casualties in crashes in rural areas. In terms of the driving environment, environmental factors such as a dark environment without streetlights, adverse weather conditions, and unfavorable terrain increase the likelihood and severity of crashes; however, they prompt drivers to take compensatory measures such as reducing speed. Insufficient lighting on rural roads delays drivers’ perception and reaction time and thus increases the severity of crashes at night (39). In particular, the likelihood of death and serious injuries due to rural road crashes that occur in the early morning (from midnight to 6:00 a.m.) is 1.6 times that in daytime or nighttime (40). A greater traffic volume on rural roads during weekdays increases the likelihood of crashes (30). The impacts of winter factors on visibility, movement space, and road surfaces are unfavorable for traffic in rural areas (41–43). Factors such as dark environments without streetlights (29), adverse weather conditions (44), and unfavorable terrain (5) lead to low visibility on rural roads, reduced vehicle braking ability, and increases in distracted driving.

Although previous studies have identified various risk factors for road traffic in rural areas, most of the research data used come from developed countries and simulation experiments. These studies often inadequately consider the economic and social conditions, the social backgrounds of drivers, and the presence of a larger number of unsafe vehicles in the rural areas of developing countries. Furthermore, it is difficult for models that include only a single variable or a small number of variables to fully explain the regional traffic risk factors for traffic crashes in rural areas. To address the limitations of the previous literature, in this study, innovative research has been conducted in the following aspects:

First, in terms of research subjects, previous literature has given little attention to the impact of the unique economic and social background of developing countries on traffic crash behaviors and serious casualties in rural areas. Associated factors include greater family financial pressures on drivers, more significant effects of traffic safety laws and regulations, less maintenance of rural roads, and more flexible agricultural working hours. These factors lead to differences between rural areas in China and those in developed countries in the influences of vehicles, roads, and time in traffic crashes in rural China.

Second, in terms of research methods, to address the issue that conclusions from traffic risk studies in urban areas may not apply to rural areas due to the urban–rural dual structure in developing countries, while considering significant urban–rural differences in the complexity of the regional traffic environment, intensity of vehicle licensing supervision, proportion of sand and gravel roads, and traffic volume, a comparative study is conducted on the occupational backgrounds of drivers, vehicle licensing statuses, road pavement types, and crash types in urban and rural areas.

Third, in terms of variable selection, many studies have overlooked the pre-existing risks inherent in driver and vehicle factors before crashes, including factors such as household registration (hukou), driver licenses, vehicle safety status, and insurance status. These factors reflect the driver’s driving ability and vehicle safety situation, which may affect at-fault traffic crash behavior and the severity of injuries and fatalities in crashes.

Fourth, in terms of innovative findings, beyond confirming known urban–rural disparities, we find that drivers’ occupations and road structures exert distinct influences on traffic safety in urban and rural areas. Specifically, while migrant workers are more likely to cause accidents in rural areas, they are more likely to cause serious injuries or fatalities in urban areas. Contrary to existing literature (35), drivers are less likely to engage in risk-compensating behavior on gravel roads in rural areas compared to urban areas, as this significantly increases the likelihood of causing accidents and resulting in serious casualties. This study unexpectedly found that drivers with rural household registration are more prone to causing major fatal accidents in rural areas. Furthermore, existing research has paid little attention to unlicensed drivers and vehicles with substandard safety conditions in rural areas. However, one-third of drivers involved in rural traffic accidents lack licenses, and 7.7% of vehicles have unfit safety status that are more likely to lead to accident-causing behaviors and result in serious casualties.

## 3 Data description and variable setting

### 3.1 Data

To ensure the authority and consistency of the data sources, the data used in this study were obtained from the Road Traffic Crash Database of the Ministry of Public Security of China, specifically, the Case Report on Specific Events by Traffic Management Departments.^4^ This database is the only officially authorized traffic database in China. The information in the database is entered by the traffic police within 24 h after completing the investigation of road traffic crash scenes and includes detailed indicators such as the driver information, severity of crash casualties, vehicle characteristics, and the road conditions and environment al factors related to the crash. Guangdong remains China’s province with the highest number of road traffic accidents. In 2024, the province recorded 37,010 road traffic accidents, resulting in 6,491 fatalities and 34,607 injuries—both figures ranking first nationwide (45). The province faces significant disparities in urban–rural development (46). In 2024, the per capita disposable income for urban residents reached 61,629 yuan, while rural residents earned only 26,729 yuan (47). By the end of 2022, counties accounted for 71.7% of Guangdong’s total area but contributed only 12.5% of the province’s GDP. In 17 counties, the per capita disposable income of farmers remained below the national average.^5^ Given that Guangdong province is “the province with the largest floating population in China,” the road user characteristics, registered residences, job categories, and other factors are deemed typical and representative (48). Thus, it is particularly relevant to take Guangdong Province as an example to conduct in-depth analysis of the risk factors influencing at-fault traffic crash behaviors and causing serious casualties in rural areas of developing countries. Therefore, 38,458 rural traffic crash reports were selected from the road traffic crash database of the Ministry of Public Security of China for crashes that occurred in Guangdong Province between 2006 and 2014. Based on these reports, we obtained information on drivers such as gender, age, hukou, driver’s license, and occupation; vehicle information including vehicle safety status, license plate status, and insurance status; road information such as road intersections, road alignment, traffic signal conditions, road physical barriers, road surface conditions, and road types; and environmental information such as terrain, lighting conditions, weather conditions, visibility, whether it was a weekend, time of accident occurrence, and season.

Following standard practices in traffic safety research, we implemented a series of data cleaning procedures. We excluded observations lacking key variable information (such as injury severity, driver liability determination, or core accident characteristics), while also removing records containing logical contradictions or invalid entries (e.g., unreasonable values for time, location, or vehicle attributes). These excluded samples constituted only a small proportion of the original data and were unlikely to systematically influence the analysis results. The final analysis sample comprised 38,458 rural accident records and 55,353 urban accident records. All subsequent analyses were conducted based on this sample.

Based on the crash liability and the severity of injuries confirmed by the traffic police, the following two dependent variables are set to determine the risk factors for the drivers’ at-fault behavior and the serious crash casualties in rural areas. The first variable is “whether the driver of the vehicle is the at-fault party causing the crash?” where 1 indicates that the driver of the vehicle bears full responsibility or the primary responsibility or the equal responsibility for the crash, and 0 indicates that the driver of the vehicle bears secondary responsibility or no responsibility for the crash. The second variable is “whether the crash causes serious casualties,” where 1 indicates serious consequences such as “death, serious injury, missing,” and 0 indicates non-serious consequences such as “minor injury, no injury.” Considering the complexity of traffic risks in rural areas and their uniqueness from those in urban areas, variables are determined for four aspects, i.e., driver factors, vehicle factors, rural road factors, and rural environmental factors, to distinguish crashes occurring in rural areas from those in rural areas, and a comparative analysis of urban and rural traffic risk factors is conducted.

This paper combines death and serious injury into a single high-severity outcome based on three primary considerations. First, within China’s traffic accident reporting system, death and serious injury are typically classified together as severe consequences, carrying similar implications and policy implications at the levels of policy response and emergency management. Both require similar preventive measures and medical interventions, a fact particularly pronounced in rural areas with limited emergency resources. Second, the transition from serious injury to death does not necessarily follow a monotonically progressive relationship. The ordering and monotonicity assumptions inherent in ordered severity models may not hold true, a problem particularly pronounced given the significant heterogeneity in medical accessibility between urban and rural areas. Third, isolating mortality outcomes leads to extremely sparse cells in some estimation samples, causing estimation instability and unreliable statistical inference. For instance, rural areas accounted for only 10% of all mortality cases, dropping to 6.97% in 2007.^6^

### 3.2 Risk factors

**Driver factors:** In the study of traffic risks among rural drivers, the demographic characteristics of drivers, such as gender, age, and education level, are important factors affecting driving behavior and crash severity (16, 49). In previous studies on traffic crashes in rural areas, 25 years of age is often used as a critical age division point (44, 50). Therefore, drivers are categorized into two groups: ≤25 years old and >25 years old. Although education, income, and social status are expected to be potential factors associated with traffic violations and accident severity, this information is generally not recorded in the traffic accident database. Instead, related information is available, as indicated by hukou origin and occupation. Two groups of the hukou household registration are examined: rural hukou and urban hukou. The variable hukou does not mean to be a proxy to determine whether a driver comes from rural area or not. It refers to the underlying difference in education and medical security level between the rural and urban hukou status (48). Driving licenses are legal credentials demonstrating drivers’ knowledge of traffic regulations and driving skills. Hence, the possession of a valid driver’s license is also considered an explanatory variable. Occupational categories are associated with drivers’ traffic risky behaviors (51). In this study, occupations are divided into farmers, general staff, workers, migrant workers, self-employed individuals, and other occupations.

**Vehicle factors:** There are a considerable number of scrapped and illegally modified vehicles in rural China (9). Drivers of unsafe vehicles are more likely to engage in dangerous driving behaviors (43). In China, the probability of death when driving unregistered vehicles is four times that of driving legally registered vehicles (18). In addition, previous studies have shown that vehicle type is an important factor affecting the severity of crash injuries in rural areas (25). Therefore, a vehicle safety status variable is set (1 = unfit; 0 = fit), a vehicle license plate variable is set (1 = without a license plate, 0 = with a license plate), and a vehicle insurance variable is set (1 = with insurance, 0 = without insurance). Vehicle types are divided into four categories: passenger vehicle, truck, motorcycle, and other vehicles.

**Road factors:** Considering that intersections significantly increase the severity of crashes in rural areas (25), and that curves and slopes are more common in rural areas (3), which significantly increase the likelihood of traffic crashes (9), an intersection variable is set (1 = yes, 0 = no), and a road alignment variable is set (1 = curve and slope, 0 = straight road). Since the lack of traffic signals (9) and road barriers (21) in rural areas may lead to more dangerous driving behavior, a traffic signal condition variable is set (1 = without traffic signal, 0 = with traffic signal), and a physical road barrier variable is set (1 = with physical road barrier, 0 = without physical road barrier). In rural areas, both unpaved roads and wet roads make it more difficult to control vehicles and cause drivers to be more cautious (25, 35). To investigate this situation, road surface condition factors are divided into three categories, namely, dry, wet, and others; and the pavement type factors are divided into four categories, namely, asphalt, cement, sand and gravel, and others.

**Environmental factors:** Previous literature has shown that the period from midnight to 6 a.m. has a significant impact on the likelihood and severity of crashes (22, 40). Differences in traffic volume lead to higher traffic risks on weekdays than on weekends (31), and the impact of winter on road traffic is greater than other seasons (43). Therefore, the day is divided into early morning (0:00–5:59), daytime (6:00–17:59) and nighttime (18:00–23:59); the week is divided into weekdays and weekends; and the year is divided according to the standards of the China Meteorological Administration into spring (March–May), summer (June–August), autumn (September–November), and winter (December–February) (52). In addition, the following environmental factors are considered: terrain conditions (5), lighting conditions (53), weather conditions (24), and visibility (7). Based on the distribution of terrain types in Guangdong Province (54), terrain factors are categorized into three types: plains, hills, and mountainous areas. Considering the important role of street lighting for traffic in rural areas, the lighting conditions are divided into three categories: daytime, nighttime with street lighting, and nighttime without street lighting, where nighttime includes two time periods, namely, early morning and night. A weather conditional variable is set (1 = bad weather and 0 = good weather). According to the definition by the China Meteorological Administration, visibility less than 200 m is defined as dense fog or heavy fog;^7^ thus, considering the relevant traffic safety requirements set out in the Regulations for the Implementation of the Road Traffic Safety Law of the People’s Republic of China,^8^ a visibility variable is also set (1 = visibility less than 200 m, and 0 = visibility greater than 200 m).

In addition, considering the impact of traffic violations and crash types on the severity of casualties in crashes in rural and urban areas, in the present study, a database is used to set variables for traffic violations and crash types, including four types of traffic violations (speeding, drunk driving, improperly overtaking, and fatigue driving) and two crash types (single-vehicle crashes and multivehicle crashes). All variable definitions and codes are detailed in Table 1.

**Table 1:** Variable definitions and descriptive statistics.

Variables	Description of variables	Rural areas	Urban areas	Full sample
		*N* = 58,987	*N* = 92,102	*N* = 151,089
Liability				
Driver at-fault	driver at-fault in the crash = 1, others = 0	41,266 (70.0%)	58,794 (63.8%)	100,060 (66.2%)
Casualties				
Fatal and serious injury	fatal and serious injury in the crash = 1, others = 0	13,467 (22.8%)	27,042 (29.4%)	40,509 (26.8%)
Human factors				
Gender				
Male	male = 1, female = 0	54,639 (92.6%)	86,514 (93.9%)	141,153 (93.4%)
Age				
≤25	≤25 = 1, others = 0	13,097 (22.2%)	16,144 (17.5%)	29,241 (19.4%)
*Hukou* origin				
Rural	rural *hukou* = 1, urban *hukou* = 0	16,559 (28.1%)	22,857 (24.8%)	39,416 (26.1%)
State of driving license				
None or not valid	having no or invalid license = 1; otherwise = 0	19,268 (32.7%)	22,350 (24.3%)	41,618 (27.5%)
Occupation				
Farmers	farmers = 1, otherwise = 0	13,457 (22.8%)	17,304 (18.8%)	30,761 (20.4%)
General staffs	general staffs = 1, otherwise = 0	2,361 (4.0%)	5,433 (5.9%)	7,794 (5.2%)
Workers	workers = 1, otherwise = 0	11,258 (19.1%)	17,619 (19.1%)	28,877 (19.1%)
Migrant workers	migrant workers = 1, otherwise = 0	10,591 (18.0%)	10,865 (11.8%)	21,456 (14.2%)
Self-employed	self-employed = 1, otherwise = 0	5,036 (8.5%)	10,963 (11.9%)	15,999 (10.6%)
Other occupations	other occupations = 1, otherwise = 0	16,284 (27.6%)	29,918 (32.5%)	46,202 (30.6%)
Vehicle factors				
Vehicle safety status				
Unfit status	unfit safety status = 1, fit safety status = 0	4,563 (7.7%)	6,054 (6.6%)	10,617 (7.0%)
License plate status				
None	without a license plate/unregistered vehicle = 1, otherwise = 0	14,095 (23.9%)	15,195 (16.5%)	29,290 (19.4%)
Insurance				
Valid	having an insurance = 1, otherwise = 0	41,227 (69.9%)	72,851 (79.1%)	114,078 (75.5%)
Vehicle type				
Passenger vehicle	passenger vehicle = 1, otherwise = 0	16,954 (28.7%)	27,769 (30.2%)	44,723 (29.6%)
Truck	truck = 1, otherwise = 0	10,838 (18.4%)	24,139 (26.2%)	34,977 (23.1%)
Motorcycle	motorcycle = 1, otherwise = 0	28,829 (48.9%)	35,649 (38.7%)	64,478 (42.7%)
Others	others = 1, otherwise = 0	2,366 (4.0%)	4,545 (4.9%)	6,911 (4.6%)
Road factors				
Intersection				
Intersection	intersection = 1, otherwise = 0	10,817 (18.3%)	14,887 (16.2%)	25,704 (17.0%)
Road alignment				
curve/slope	curve/slope = 1, smooth = 0	48,327 (81.9%)	74,270 (80.6%)	122,597 (81.1%)
Traffic signal conditions				
None	none = 1, otherwise = 0	20,327 (34.5%)	12,166 (13.2%)	32,493 (21.5%)
Whether there are physical barriers in roads				
Physical barriers	having physical barriers = 1, otherwise = 0	17,657 (29.9%)	60,419 (65.6%)	78,076 (51.7%)
Road surface				
Dry	dry = 1, otherwise = 0	52,211 (88.5%)	79,812 (86.7%)	132,023 (87.4%)
Wet	wet = 1, otherwise = 0	5,592 (9.5%)	10,467 (11.4%)	16,059 (10.6%)
Others	others = 1, otherwise = 0	1,184 (2.0%)	1823 (2.0%)	3,007 (2.0%)
Road structure				
Pitch	pitch road = 1, otherwise = 0	12,975 (22.0%)	33,256 (36.1%)	46,231 (30.6%)
Cement	cement road = 1, otherwise = 0	44,797 (75.9%)	57,888 (62.9%)	102,685 (68.0%)
Gravel	gravel road = 1, otherwise = 0	495 (0.8%)	86 (0.1%)	581 (0.4%)
Others	other road = 1, otherwise = 0	720 (1.2%)	872 (0.9%)	1,592 (1.1%)
Environmental factors				
Terrain				
Plain	plain = 1, otherwise = 0	14,374 (24.4%)	27,090 (29.4%)	41,464 (27.4%)
Hill	hill = 1, otherwise = 0	38,184 (64.7%)	49,710 (54.0%)	87,894 (58.2%)
Mountain	mountain = 1, otherwise = 0	6,429 (10.9%)	15,302 (16.6%)	21,731 (14.4%)
Street-light condition				
Daylight	daylight = 1, otherwise = 0	37,174 (63.0%)	52,377 (56.9%)	89,551 (59.3%)
Dark but lighted	In the night with street-light = 1, otherwise = 0	13,153 (22.3%)	22,231 (24.1%)	35,384 (23.4%)
Dark	In the night without street-light = 1, otherwise = 0	7,812 (13.2%)	16,102 (17.5%)	23,914 (15.8%)
Weather condition				
Bad	bad weather = 1, good weather = 0	13,501 (22.9%)	22,825 (24.8%)	36,326 (24.0%)
Visibility level				
Bad	bad visibility = 1, good visibility = 0	37,126 (62.9%)	54,517 (59.2%)	91,643 (60.7%)
Day of a week				
Weekend	weekend = 1, otherwise = 0	16,616 (28.2%)	25,408 (27.6%)	42,024 (27.8%)
Time of day				
0:00–5:59	0:00–5:59 = 1, otherwise = 0	4,818 (8.2%)	12,933 (14.0%)	17,751 (11.7%)
6:00–17:59	6:00–17:59 = 1, otherwise = 0	37,471 (63.5%)	52,742 (57.3%)	90,213 (59.7%)
18:00–23:59	18:00–23:59 = 1, otherwise = 0	16,698 (28.3%)	26,427 (28.7%)	43,125 (28.5%)
Season				
Spring	spring = 1, otherwise = 0	14,009 (23.7%)	22,650 (24.6%)	36,659 (24.3%)
Summer	summer = 1, otherwise = 0	19,930 (33.8%)	30,702 (33.3%)	50,632 (33.5%)
Autumn	autumn = 1, otherwise = 0	10,856 (18.4%)	15,827 (17.2%)	26,683 (17.7%)
Winter	winter = 1, otherwise = 0	14,192 (24.1%)	22,923 (24.9%)	37,115 (24.6%)
Crash condition				
Crash type				
Single-vehicle collision	single-vehicle collision = 1, otherwise = 0	3,122 (5.3%)	4,773 (5.2%)	7,895 (5.2%)
Multiple-vehicle collision	multiple-vehicle collision = 1, otherwise = 0	46,886 (79.5%)	77,579 (84.2%)	124,465 (82.4%)
Traffic violation				
Speeding	speeding = 1, otherwise = 0	1,106 (1.9%)	2,504 (2.7%)	3,610 (2.4%)
Drunk driving	drunk driving = 1, otherwise = 0	1,000 (1.7%)	1,150 (1.2%)	2,150 (1.4%)
Improper overtaking	improper overtaking = 1, otherwise = 0	1,602 (2.7%)	2,671 (2.9%)	4,273 (2.8%)
Fatigue driving	fatigue driving = 1, otherwise = 0	194 (0.3%)	658 (0.7%)	852 (0.6%)

We acknowledge that the model cannot exhaustively account for all potential confounding factors, particularly the influence of unobservable variables. However, by systematically controlling key determinants such as human factors, vehicles, roads, environment, and violations, this study minimizes the risk of omitted variable bias at the observable level. Furthermore, given the significant differences in the distribution of these factors between rural and urban areas in China, incorporating them into the model facilitates analysis of their respective impacts on accident severity across these settings. Additionally, these influencing factors have been extensively documented in prior studies on traffic accident determinants (48, 55), enabling meaningful cross-scenario comparisons.

### 3.3 Method

Studies investigating factors influencing the severity of traffic accidents often employ models such as standard Logit, random-effects Logit, multilevel Logit, and mixed Logit. However, this paper aims to identify average risk factors with policy implications rather than characterize the distribution characteristics of individual-level latent preferences or unobserved heterogeneity. Furthermore, the traffic accident data employed in this study consists of cross-sectional police reports lacking repeated observations of the same drivers, vehicles, or road segments. This inherently limits the feasibility and interpretive value of random-effects Logit, multilevel Logit, or mixed-effects models.^9^ It should be noted that alternative models have applicability boundaries: multilevel or mixed-effects models typically suit data with clear hierarchical structures or longitudinal tracking, while rare-event Logit models primarily target events with extremely low occurrence probabilities. However, serious injury/fatality accidents in this study’s sample are not extremely rare. Therefore, following Zhang et al. (48) and Peek-Asa et al. (7), we adopt the standard Logit model as our baseline.^10^

We primarily investigate risk factors for traffic violations and serious injury or fatality accidents in rural areas, while also examining risk factors in urban areas and differences in risk factors between urban and rural areas. All models are estimated separately for rural and urban subsamples to allow the full set of coefficients to vary across contexts, rather than imposing homogeneous effects through interaction terms. The specific model is set up as follows:


(1) $$\ln(\frac{P_{i}}{1-P_{i}})=\alpha +\beta_{i}*X_{i}+\mu_{i}$$

where $P_{i}$ is the probability of drivers in rural areas causing crashes or experiencing serious injuries in crashes, $1-P_{i}$ is the probability that drivers in rural areas do not cause crashes or experience less serious casualties in crashes, $\beta_{i}$ is the coefficient to be estimated, $\mu_{i}$ is an error term, and $X_{i}$ is a risk factor influencing drivers in rural areas causing crashes or experiencing serious injuries in crashes. For ease of interpretation, we use the odds ratio (OR) to analyze the results. When estimating the factors influencing the severity of injuries or fatalities in accidents caused by drivers in urban areas, the model settings and equations are identical to those in Equation 1, with the only modification being the replacement of “rural areas” with “urban areas.”

### 3.4 Variable descriptions and descriptive statistics

Our model incorporates numerous risk factors, but multicollinearity among these variables may compromise the validity of estimates. Therefore, we calculated the variance inflation factor (VIF). The results show that the average VIF for the model with “whether the driver was at fault” as the dependent variable is 2.59, and the average VIF for the model with “whether serious casualties occurred” as the dependent variable is 2.39. Both values are significantly below the commonly used threshold (generally 10), indicating that the models do not exhibit severe multicollinearity issues.

Table 1 presents the variable descriptions and descriptive statistics. In rural areas, 70.0% of traffic crashes involve at-fault behavior, higher than the proportion in urban areas (63.8%). In rural areas, 22.8% of traffic crashes result in serious casualties, lower than the proportion in urban areas (29.4%).

Regarding the characteristics of drivers in rural areas, the vast majority of drivers in rural areas (92.6%) and urban areas (93.9%) are male, and the proportion of young drivers ≤25 years old in rural areas (22.2%) is higher than that in urban areas (17.5%). As expected, the proportion of drivers without a valid driver’s license in rural areas (32.7%) is higher than that in urban areas (24.3%). However, contrary to common belief, there is little difference between the proportion of drivers with rural hukou in rural areas (28.1%) and that in urban areas (24.8%). In rural areas compared to urban areas, the order of driver occupations by population proportion is as follows, from highest to lowest: migrant workers (18.0% vs. 11.8%), farmers (22.8% vs. 18.8%), self-employed individuals (8.5% vs. 11.9%), general staff (4.0% vs. 5.9%), and workers (19.1% vs. 19.1%).

In terms of vehicle factors in rural areas, there are more unsafe vehicles (7.7% vs. 6.6%) and more unregistered vehicles (23.9% vs. 16.5%) in rural areas than in urban areas. The proportion of vehicles with insurance in rural areas is 69.9%, which is lower than that in urban areas (79.1%). Motorcycles are the means of transportation most likely to be involved in crashes, accounting for 48.9% of the total number of crash vehicles in rural areas and ranking first in urban areas, accounting for 38.7%. Crashes involving passenger vehicles and trucks occur more frequently in urban areas, with 30.2% of urban area crashes and 28.7% of rural area crashes involving passenger vehicles, and 26.2% of urban area crashes and 18.4% of rural area crashes involving trucks.

Regarding road factors in rural areas, 18.3% of crashes in rural areas and 16.2% of crashes in urban areas occur at intersections. Curves and slopes affect the majority of crashes in rural areas (81.9%) and in urban areas (80.6%). Notably, on the one hand, the number of crashes involving missing traffic signals in rural areas reaches 34.5%, which is much higher than the 13.2% in urban areas. On the other hand, only 29.9% of crashes in rural areas involve physical road barriers, which is far lower than the 65.6% in urban areas. Most of the crashes in rural areas (88.5%) and in urban areas (86.7%) occur on dry roads. Cement is the road pavement type that cause the most traffic crashes in rural areas (75.9%) and urban areas (62.9%), while sand and gravel is the pavement type that has the least impact on crashes in rural areas (0.8%) and urban areas (0.1%).

In terms of environmental factors in rural areas, more than half of the crashes in rural areas (64.7%) and in urban areas (54.0%) occur in hilly terrain. Notably, more than half of the traffic crashes in rural areas and urban areas occur during the day, accounting for 63.0 and 56.9%, respectively. In rural areas, there are more traffic crashes at night with street lighting than at night without street lighting (22.3% vs. 13.2%), and this trend is also observed in urban areas (24.1% vs. 17.5%). Adverse weather conditions are associated with 22.9% of crashes in rural areas and 24.8% of crashes in urban areas; and poor visibility conditions are associated with 62.9% of crashes in rural areas and 59.2% of crashes in urban areas. During the day, more traffic crashes occur in rural areas (63.5%) and urban areas (57.3%) during the time period of 6:00 to 17:59 than during the other 12 h. Moreover, traffic crashes are more likely to occur during the time period from 18:00 to 23:59 than during the time period from 0:00 to 5:59 in rural areas (28.3% vs. 8.2%) and urban areas (28.7% vs. 14.0%). During the week, 28.2% of the crashes in rural areas and 27.6% of the crashes in urban areas occur on weekends. During the year, summer has the greatest impact on rural areas (33.8%) and urban areas (33.3%), and autumn has the least impact on rural areas (18.4%) and urban areas (17.2%). Among the crash condition factors, single-vehicle crashes account for 5.3 and 5.2% of the crashes in rural areas and urban areas, respectively, and multivehicle crashes account for 79.5 and 84.2%, respectively. Improper passing is a major traffic violation in both rural and urban areas, accounting for 2.7 and 2.9%, respectively, of crashes, while fatigue driving is a minor traffic violation, accounting for 0.3 and 0.7% of crashes in rural and urban areas, respectively.

To assess the relationship between risk factors and at-fault crash behavior, we conducted Pearson chi-square tests to examine the variables influencing at-fault behavior in traffic crashes in rural areas and urban areas. The results are presented in Columns (1) and (2) of Table 2. The test results for rural areas show that driver factors (except driver’s license), vehicle factors, road factors (except road alignment), and environmental factors (except terrain, weekend, and season) are significantly correlated with drivers’ at-fault behavior in rural areas. For urban areas, driver factors (except driver’s license), vehicle factors, road factors (except road alignment and road surface conditions), and environmental factors (except terrain, weather conditions and weekends) are significantly correlated with driver’s at-fault behavior in urban areas.

**Table 2:** Chi-square test of independence for driver at-fault crashes.

Variables	(1)	(2)
	Rural areas	Urban areas
	x^2^(*p*-value), df	x^2^(*p*-value), df
Human factors		
Gender	101.259^***^(<0.001), 1	108.228^***^(<0.001), 1
Age	101.246^***^(<0.001), 1	111.858^***^(<0.001), 1
*Hukou* origin	137.796^***^(<0.001), 1	96.875^***^(<0.001), 1
State of driving license	1.169 (0.280), 1	1.058 (0.304), 1
Occupation	147.375^***^(<0.001), 5	56.252^***^(<0.001), 5
Vehicle factors		
Vehicle safety status	4.461^**^(0.035), 1	124.001^***^(<0.001), 1
License plate status	37.963^***^(<0.001), 1	67.254^***^(<0.001), 1
Insurance	58.454^***^(<0.001), 1	81.124^***^(<0.001), 1
Vehicle type	834.231^***^(<0.001), 3	968.512^***^(<0.001), 3
Road factors		
Intersection	22.052^***^(<0.001), 1	11.525^***^(0.001), 1
Road alignment	2.061 (0.151), 1	0.502 (0.479), 1
Traffic signal conditions	43.671^***^(<0.001), 1	46.796^***^(<0.001), 1
Whether there are physical barriers in roads	34.486^***^(<0.001), 1	67.695^***^(<0.001), 1
Road surface	4.970^*^(0.083), 2	3.714 (0.156), 2
Road structure	74.015^***^(<0.001), 3	203.268^***^(<0.001), 3
Environmental factors		
Terrain	0.010 (0.995), 2	2.603 (0.272), 2
Street-light condition	134.624^***^(<0.001), 2	54.192^***^(<0.001), 2
Weather condition	4.389^**^(0.036), 1	1.254 (0.263), 1
Visibility level	36.184^***^(<0.001), 1	40.427^***^(<0.001), 1
Day of a week	0.331 (0.565), 1	0.347 (0.556), 1
Time of day	124.611^***^(<0.001), 2	78.969^***^(<0.001), 2
Season	2.279 (0.517), 3	6.960^*^(0.073), 3

*p < 0.1; **p < 0.05; ***p < 0.01.

To further assess the relationship between the risk factors and the severity of injuries, Pearson chi-square tests were conducted to examine the relevant variables influencing serious casualties in rural areas and urban areas, and the results are shown in Columns (1) and (2) of Table 3, respectively. For rural areas, driver factors (except age), vehicle factors, road factors, environmental factors (except weather conditions), and crash condition factors are significantly correlated with serious casualties caused by crashes in rural areas. The chi-square test results for urban areas show that driver factors, vehicle factors (excluding license plate status), road factors, environmental factors (excluding visibility), and crash condition factors are significantly correlated with serious casualties caused by crashes in urban areas.

**Table 3:** Chi-square test of independence for crash severity.

Variables	(1)	(2)
	Rural areas	Urban areas
	x^2^(*p*-value), df	x^2^(*p*-value), df
Human factors		
Gender	124.873^***^(<0.001), 1	246.178^***^(<0.001), 1
Age	0.178 (0.673), 1	3.409^*^(0.065), 1
*Hukou* origin	288.823^***^(<0.001), 1	328.458^***^(<0.001), 1
State of driving license	105.911^***^(<0.001), 1	11.262^***^(0.001), 1
Occupation	328.603^***^(<0.001), 5	199.752^***^(<0.001), 5
Vehicle factors		
Vehicle safety status	234.715^***^(<0.001), 1	417.094^***^(<0.001), 1
License plate status	66.326^***^(<0.001), 1	2.643 (0.104), 1
Insurance	186.310^***^(<0.001), 1	88.183^***^(<0.001), 1
Vehicle type	636.678^***^(<0.001), 3	1.1e+03^***^(<0.001), 3
Road factors		
Intersection	18.695^***^(<0.001), 1	177.564^***^(<0.001), 1
Road alignment	360.010^***^(<0.001), 1	605.999^***^(<0.001), 1
Traffic signal conditions	14.858^***^(<0.001), 1	140.172^***^(<0.001), 1
Whether there are physical barriers in roads	26.388^***^(<0.001), 1	28.5117^***^(<0.001), 1
Road surface	24.986^***^(<0.001), 2	70.629^***^(<0.001), 2
Road structure	52.150^***^(<0.001), 3	20.404^***^(<0.001), 3
Environmental factors		
Terrain	465.722^***^(<0.001), 2	712.021^***^(<0.001), 2
Street-light condition	487.559^***^(<0.001), 2	1.8e+03^***^(<0.001), 2
Weather condition	0.134 (0.715), 1	4.628^**^(0.031), 1
Visibility level	22.006^***^(<0.001), 1	0.570 (0.450), 1
Day of a week	3.476^*^(0.062), 1	11.444^***^(0.001), 1
Time of day	528.811^***^(<0.001), 2	1.3e+03^***^(<0.001), 2
Season	26.599^***^(<0.001), 3	53.582^***^(<0.001), 3
Crash condition		
Crash type	464.343^***^(<0.001), 1	180.445^***^(<0.001), 1
Traffic violation	132.110^***^(<0.001), 3	153.779^***^(<0.001), 3

*p < 0.1; **p < 0.05; ***p < 0.01.

## 4 Empirical results

Table 4 compares the factors influencing driver-related accident behaviors in rural versus urban areas. Table 5 further compares the factors influencing accident severity and fatalities in rural versus urban areas, with the reference group for each variable highlighted as “base.” To assess the discriminative performance of the Logit models, we also calculated the ROC curves (Appendix Figures A1, A2) and the corresponding area under the ROC curve (AUC). Results indicate that across both rural and urban samples, all baseline Logit models achieved acceptable discriminatory capability in distinguishing between at-fault and non-at-fault accidents, as well as severe versus non-severe accidents, thereby enabling effective risk identification.

**Table 4:** Factors related to driver at-fault crashes.

Variables	(1)	(2)
	Rural areas	Urban areas
Human factors		
Gender (base: female)	1.227^***^	1.226^***^
	(0.042)	(0.035)
Age (base: > 25)	1.292^***^	1.277^***^
	(0.030)	(0.025)
*Hukou* origin (base: urban)	0.783^***^	0.827^***^
	(0.017)	(0.015)
State of driving license (base: valid license)	1.429^***^	1.364^***^
	(0.038)	(0.031)
Occupation (base: farmers)		
General staffs	0.962	0.936^*^
	(0.050)	(0.033)
Workers	0.976	0.935^***^
	(0.030)	(0.023)
Migrant workers	1.197^***^	1.147^***^
	(0.038)	(0.032)
Self-employed	0.948	0.856^***^
	(0.036)	(0.023)
Others	1.019	0.925^***^
	(0.028)	(0.020)
Vehicle factors		
Vehicle safety status (base: fit)	1.094^**^	1.413^***^
	(0.038)	(0.042)
License plate status (base: with a license plate)	1.021	0.956^*^
	(0.028)	(0.023)
Insurance (base: invalid)	0.976	1.037^*^
	(0.022)	(0.020)
Vehicle type (base: motorcycle)		
Passenger vehicle	2.259^***^	1.953^***^
	(0.060)	(0.040)
Truck	2.148^***^	1.761^***^
	(0.064)	(0.037)
Others	1.558^***^	1.173^***^
	(0.075)	(0.040)
Road factors		
Intersection (base: no)	0.907^***^	0.944^***^
	(0.021)	(0.018)
Road alignment (base: smooth)	1.028	1.008
	(0.026)	(0.019)
Traffic signal conditions (base: having traffic signal)	1.172^***^	1.150^***^
	(0.025)	(0.025)
Whether there are physical barriers in roads (base: no)	0.922^***^	0.920^***^
	(0.020)	(0.015)
Road surface (base: dry)		
Wet	0.956	0.946^**^
	(0.036)	(0.026)
Others	1.059	1.032
	(0.072)	(0.053)
Road structure (base: pitch)		
Cement	1.192^***^	1.256^***^
	(0.027)	(0.020)
Gravel	1.507^***^	1.217
	(0.166)	(0.281)
Others	1.355^***^	0.989
	(0.121)	(0.073)
Environmental factors		
Terrain (base: plain)		
Hill	1.020	1.029^*^
	(0.022)	(0.017)
Mountain	1.068^*^	1.005
	(0.038)	(0.023)
Street-light condition (base: daylight)		
Dark but lighted	1.050	1.030
	(0.091)	(0.065)
Dark	1.161^*^	0.955
	(0.102)	(0.060)
Weather condition (base: good)	1.038	0.982
	(0.027)	(0.020)
Visibility level (base: good)	1.035^*^	1.043^***^
	(0.020)	(0.016)
Weekends (base: weekdays)	1.001	0.985
	(0.020)	(0.015)
Time of day (base: 0:00–5:59)		
6:00–17:59	0.927	1.023
	(0.084)	(0.065)
18:00–23:59	1.033	1.147^***^
	(0.039)	(0.026)
Season (base: summer)		
Spring	0.973	1.002
	(0.024)	(0.019)
Autumn	1.024	1.043^**^
	(0.027)	(0.022)
Winter	0.974	1.021
	(0.024)	(0.019)
Pseudo R-squared	0.027	0.019
AUC	0.612	0.595
*N*	58,987	92,102

Exponentiated coefficients; Standard errors in parentheses. Base indicates the reference group. ^*^p < 0.1, ^**^p < 0.05, ^***^p < 0.01.

**Table 5:** Factors related to fatal and serious injury in crashes.

Variables	(1)	(2)
	Rural areas	Urban areas
Human factors		
Gender (base: female)	1.354^***^	1.387^***^
	(0.060)	(0.049)
Age (base: > 25)	0.970	0.966^*^
	(0.024)	(0.020)
*Hukou* origin (base: urban)	1.219^***^	1.239^***^
	(0.029)	(0.023)
State of driving license (base: valid license)	1.203^***^	1.230^***^
	(0.036)	(0.030)
Occupation (base: farmers)		
General staffs	1.055	1.020
	(0.059)	(0.038)
Workers	0.755^***^	1.007
	(0.026)	(0.027)
Migrant workers	0.824^***^	1.082^***^
	(0.029)	(0.032)
Self-employed	0.900^**^	1.156^***^
	(0.038)	(0.033)
Others	0.945^*^	1.022
	(0.028)	(0.024)
Vehicle factors		
Vehicle safety status (base: fit)	1.466^***^	1.504^***^
	(0.051)	(0.043)
License plate status (base: with a license plate)	1.039	0.921^***^
	(0.031)	(0.024)
Insurance (base: invalid)	0.818^***^	0.802^***^
	(0.021)	(0.017)
Vehicle type (base: motorcycle)		
Passenger vehicle	1.014	1.073^***^
	(0.032)	(0.025)
Truck	1.825^***^	1.592^***^
	(0.058)	(0.037)
Others	1.842^***^	1.867^***^
	(0.089)	(0.066)
Road factors		
Intersection (base: no)	0.996	0.872^***^
	(0.027)	(0.019)
Road alignment (base: smooth)	0.758^***^	0.742^***^
	(0.021)	(0.015)
Traffic signal conditions (base: having traffic signal)	0.834^***^	0.751^***^
	(0.019)	(0.018)
Whether there are physical barriers in roads (base: no)	1.237^***^	1.016
	(0.030)	(0.018)
Road surface (base: dry)		
Wet	1.117^***^	1.255^***^
	(0.045)	(0.036)
Others	1.387^***^	1.110^*^
	(0.096)	(0.062)
Road structure (base: pitch)		
Cement	1.036	0.956^***^
	(0.027)	(0.016)
Gravel	1.401^***^	0.573^**^
	(0.146)	(0.156)
Others	0.736^***^	0.609^***^
	(0.078)	(0.051)
Environmental factors		
Terrain (base: plain)		
Hill	0.938^***^	0.941^***^
	(0.023)	(0.017)
Mountain	1.375^***^	1.288^***^
	(0.050)	(0.030)
Street-light condition (base: daylight)		
Dark but lighted	1.006	1.113
	(0.096)	(0.075)
Dark	1.478^***^	1.815^***^
	(0.141)	(0.123)
Weather condition (base: good)	0.923^***^	0.873^***^
	(0.027)	(0.019)
Visibility level (base: good)	1.001	0.887^***^
	(0.022)	(0.014)
Weekends (base: weekdays)	1.021	1.054^***^
	(0.023)	(0.018)
Time of day (base: 0:00–5:59)		
6:00–17:59	0.586^***^	0.717^***^
	(0.057)	(0.049)
18:00–23:59	0.589^***^	0.677^***^
	(0.022)	(0.016)
Season (base: summer)		
Spring	1.014	0.988
	(0.028)	(0.020)
Autumn	1.085^***^	1.084^***^
	(0.032)	(0.024)
Winter	1.119^***^	1.137^***^
	(0.030)	(0.022)
Crash condition		
Crash type		
Single-vehicle collision (base: others)	1.658^***^	0.898^***^
	(0.078)	(0.035)
Multiple-vehicle collision (base: others)	0.879^***^	0.707^***^
	(0.025)	(0.017)
Traffic violation		
Speeding (base: others)	2.483^***^	1.809^***^
	(0.159)	(0.077)
Drunk driving (base: others)	1.461^***^	1.661^***^
	(0.105)	(0.104)
Improper overtaking (base: others)	1.115^*^	1.075
	(0.069)	(0.048)
Fatigue driving (base: others)	1.240	1.431^***^
	(0.197)	(0.118)
Pseudo R-squared	0.051	0.049
AUC	0.656	0.653
*N*	58,987	92,102

Exponentiated coefficients; Standard errors in parentheses. Base indicates the reference group. ^*^p < 0.1, ^**^p < 0.05, ^***^p < 0.01.

### 4.1 Analysis of the factors influencing the at-fault behavior of traffic crashes in rural areas

Column (1) of Table 4 shows the risk factors for drivers in rural areas associated with at-fault behavior. First, gender, age, hukou, driver’s license, and occupation of migrant workers are significant factors among driver factors in rural areas. In rural areas, male drivers (OR = 1.227), drivers aged ≤ 25 years old (OR = 1.292), and drivers without a driver’s license (OR = 1.429) are more likely to exhibit at-fault behavior. Compared with drivers with urban hukou, drivers with rural hukou are less likely to cause at-fault crashes in rural areas (OR = 0.783). Among the drivers of various occupations in rural areas, migrant workers are more likely than farmers (OR = 1.197) to exhibit at-fault behavior. Second, among the vehicle factors, vehicle safety status and vehicle type significantly affect the at-fault behavior of drivers in rural areas. Vehicles with an unfit safety status (OR = 1.094) are more likely to cause at-fault behavior. Notably, compared to motorcycles, passenger vehicles (OR = 2.259) and trucks (OR = 2.148) are more likely to cause at-fault crashes in rural areas. Third, many road factors, including intersections, traffic signal conditions, physical road barriers, and pavement type, have significant relationships with at-fault traffic crashes in rural areas. The OR of the intersection variable is 0.907, indicating that drivers are more likely to cause at-fault crashes on non-intersection roads in rural areas. Unsurprisingly, at-fault behaviors are more likely to occur in the absence of traffic signals (OR = 1.172), while at-fault behaviors are less likely to occur in the presence of physical road barriers (OR = 0.922). Compared with those on asphalt roads, drivers on cement (OR = 1.192) and sand and gravel (OR = 1.507) roads are more likely to cause at-fault crashes. Fourth, terrain, lighting conditions, and visibility are the significant environmental factors in rural areas. Compared with plain terrain, mountainous terrain (OR = 1.068) is more likely to lead drivers to cause at-fault crashes in rural areas. Among the lighting condition factors, nighttime without street lighting (OR = 1.161) is more likely than that the daytime to cause at-fault behavior. Under poor visibility conditions (OR = 1.035), drivers in rural areas are more likely to cause at-fault crashes.

Column (2) of Table 4 shows the risk factors for drivers in urban areas to exhibit at-fault behavior. First, regarding driver factors, male drivers (OR = 1.226), drivers aged ≤25 years old (OR = 1.277), and drivers without a valid license (OR = 1.364) are more likely to cause at-fault crashes in urban areas, while drivers with rural hukou (OR = 0.827) are less likely to cause at-fault crashes in urban areas. Compared with farmers, migrant workers (OR = 1.147) are more likely to cause at-fault crashes in urban areas, and drivers who are general staff (OR = 0.936), workers (OR = 0.935), and self-employed individuals (OR = 0.856) are less likely to cause at-fault crashes. Second, in terms of vehicle factors in urban areas, vehicles with an unfit safety status (OR = 1.413) are more likely to cause at-fault crashes. Notably, vehicles without a license plate show OR = 0.956, and vehicles with insurance show OR = 1.037, indicating that vehicles with license plates and vehicles with insurance in urban areas are more likely to cause at-fault crashes. Compared with motorcycles, both buses (OR = 1.953) and trucks (OR = 1.761) are more likely to cause at-fault crashes in urban areas. Third, considering the road factors in urban areas, at-fault behaviors are less likely to occur in urban areas at intersections (OR = 0.944) and roads with physical road barriers (OR = 0.920) and more likely to occur in the absence of traffic signals (OR = 1.150). In urban areas, at-fault behaviors are less likely to occur on roads with wet surfaces (OR = 0.946) but more likely to occur on roads with concrete pavement (OR = 1.256). Fourth, in terms of environmental factors in urban areas, at-fault behaviors are more likely to occur in hilly terrain (OR = 1.029) and under poor visibility (OR = 1.043). Drivers in urban areas are more likely to exhibit at-fault behavior during the time period of 18:00–23:59 (OR = 1.147) than during the time period of 0:00–5:59 and are more likely to exhibit at-fault behavior in autumn (OR = 1.043) than in summer.

### 4.2 Analysis of the factors influencing the severity of casualties in traffic crashes in rural areas

Column (1) of Table 5 shows the risk factors for serious casualties in crashes in rural areas. First, among driver factors, gender, hukou, driver’s license, and occupation significantly affect the severity of crashes in rural areas. Male drivers (OR = 1.354), drivers with rural hukou (OR = 1.219), and drivers without a valid license (OR = 1.203) are more likely to cause serious casualties in rural areas. Compared with farmers, drivers who are workers (OR = 0.755), migrant workers (OR = 0.824), and self-employed individuals (OR = 0.900) are less likely to cause serious casualties. Second, among vehicle factors in rural areas, vehicle safety status, insurance, and vehicle type are significant factors that affect the severity of crashes in rural areas. Vehicles with an unfit safety status (OR = 1.466) are more likely to cause serious casualties, while vehicles with insurance (OR = 0.818) are much less likely to cause serious casualties. Among the various vehicles driven in rural areas, trucks (OR = 1.825) are more likely than motorcycles to cause serious casualties. Third, among rural road factors, road alignment, traffic signal conditions, physical road barriers, road surface conditions, and pavement type significantly affect the severity of crashes in rural areas. In rural areas, non-straight road alignment (OR = 0.758) and the absence of traffic signals (OR = 0.834) are associated with low risks of serious casualties; however, the existence of physical road barriers (OR = 1.237) increases the likelihood of severe casualties. In rural areas, wet road surfaces (OR = 1.117) are more likely to cause serious casualties than dry road surfaces, and sand and gravel pavement (OR = 1.401) is more likely to cause serious casualties than asphalt pavement. Fourth, among the environmental factors in rural areas, terrain, lighting conditions, weather conditions, time of day, and season are significant factors affecting the severity of crashes in rural areas. Compared with plains, hilly terrain (OR = 0.938) has a lower chance of causing serious casualties, while mountainous terrain (OR = 1.375) has a higher chance of causing serious casualties. In rural areas, serious casualties are more likely to occur at night without street lighting (OR = 1.478) than during the day, however, serious casualties are less likely to occur in poor weather conditions (OR = 0.923) than under good weather conditions. Compared to the time period of 0:00–5:59, the time periods of 6:00–17:59 (OR = 0.586) and 18:00–23:59 (OR = 0.589) are less likely to cause serious casualties. The risk of serious injuries occurring in rural areas in autumn (OR = 1.085) and winter (OR = 1.119) is greater than that in summer. In addition, regarding the crash condition factors, single-vehicle crashes (OR = 1.658) have a greater impact on the severity of crashes in rural areas, while multivehicle crashes (OR = 0.879) have a lesser impact. Among various traffic violations, speeding (OR = 2.483), drunk driving (OR = 1.461), and improper overtaking (OR = 1.115) significantly increase the risk of serious casualties in crashes.

**Figure 1:**
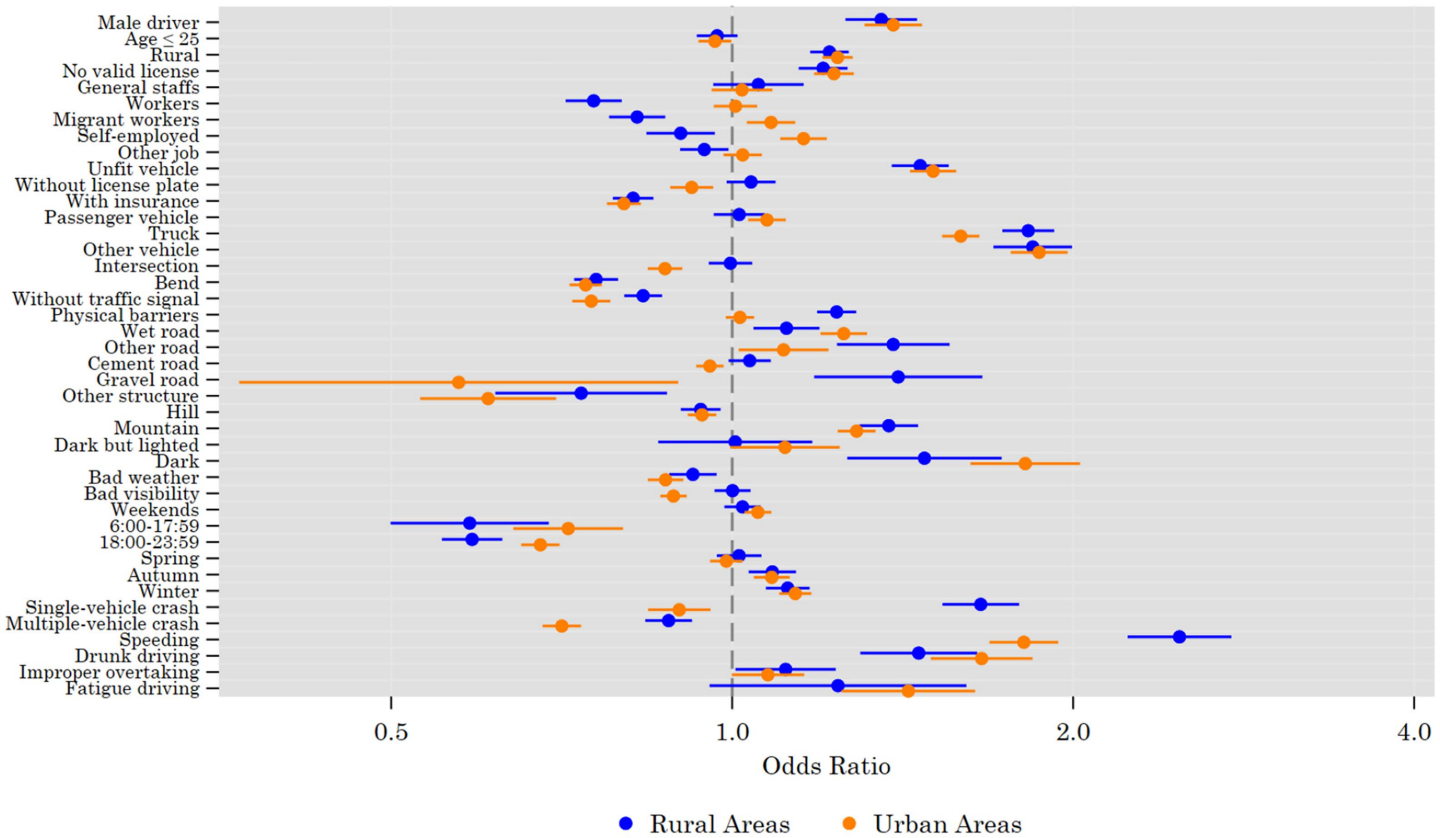
Forest plot of odds ratios for severe crash outcomes.

Column (2) of Table 5 shows the risk factors for serious casualties in crashes in urban areas. First, concerning driver factors in urban areas, male drivers (OR = 1.387), drivers with rural hukou (OR = 1.239) and drivers without a valid driver’s license (OR = 1.230) are more likely to cause serious casualties, but drivers aged ≤25 years old (OR = 0.966) are more likely to cause serious casualties. Both migrant workers (OR = 1.082) and self-employed individuals (OR = 1.156) are more likely than farmers to cause serious casualties in urban areas. Second, in terms of vehicle factors, vehicles with an unfit safety status (OR = 1.504) are likely to cause serious casualties. However, vehicles without license plates (OR = 0.921) and those with insurance (OR = 0.802) have a low risk of causing serious casualties. In urban areas, passenger vehicles (OR = 1.073) and trucks (OR = 1.592) are more likely than motorcycles to cause serious casualties. Third, in terms of urban road factors, crashes occurring at intersections (OR = 0.872), curves and slopes (OR = 0.742), and in the absence of traffic signals (OR = 0.751) are all less likely to cause serious casualties. The risk of serious casualties occurring on wet urban roads (OR = 1.255) is greater than that on dry roads. Compared with asphalt pavement, both cement roads (OR = 0.956) and sand and gravel roads (OR = 0.573) are less likely to cause serious casualties in urban areas. Fourth, many environmental factors affect the risk of serious casualties in crashes in urban areas. Compared with those in plains, serious casualties in crashes in urban areas are less likely to occur in hilly terrain (OR = 0.941) but more likely to occur in mountainous terrain (OR = 1.288). Nighttime without street lighting (OR = 1.815) is more likely than daytime without street lighting to cause crashes with serious casualties in urban areas. The likelihood of serious casualties caused by adverse weather conditions (OR = 0.873) and poor visibility (OR = 0.887) is low, while the likelihood of serious casualties during weekends (OR = 1.054) is higher. The likelihood of serious casualties in urban areas is low during the time periods of 7:00–17:59 (OR = 0.717) and 18:00–23:59 (OR = 0.677) but high in autumn (OR = 1.084) and winter (OR = 1.137). In addition, we consider the impact of crash conditions on the occurrence of serious casualties caused by crashes. On the one hand, compared with that in other crash types in urban areas, the risk of serious casualties in single-vehicle crashes (OR = 0.898) and multivehicle crashes (OR = 0.707) is low. On the other hand, compared with other traffic violations in urban areas, speeding (OR = 1.809), drunk driving (OR = 1.661), and fatigue driving (OR = 1.431) are more likely to cause crashes with serious casualties. To more intuitively illustrate the direction and magnitude of each risk factor’s impact in rural versus urban areas, thereby facilitating comparisons of urban–rural heterogeneity, we present a forest plot in Figure 1 showing the ORs and their 90% confidence intervals corresponding to Table 5.

## 5 Discussion and conclusion

Using the logit regression method, we list the risk factors that influence drivers’ causing crashes and serious casualties in rural areas in Tables 4, 5, respectively. The differences and correlations between the two are further compared and analyzed below. In summary, simultaneous factors worth noting that significantly increase the likelihood of drivers causing at-fault crashes and serious injuries in rural areas include being male, having no valid driver’s license, and driving unsafe vehicles. In addition, trucks are more dangerous than motorcycles; sand and gravel roads are more dangerous than asphalt roads; mountainous terrain is more dangerous than plain terrain; and nighttime without street lighting is more dangerous than daytime. Some risk factors have opposite effects on causing drivers to have at-fault crashes and serious casualties. For example, migrant workers are more likely than farmers to cause at-fault crashes but are less likely to cause serious casualties. The absence of traffic signals increases the likelihood of at-fault behavior but reduces the likelihood of serious casualties.

In contrast, when drivers with rural hukou and physical road barriers are involved, at-fault crashes are less likely to occur, but if a crash does occur, it is more likely to cause serious casualties. In addition, some factors increase the risk of at-fault crashes without affecting the severity of casualties, including drivers aged ≤25 years old and poor visibility environmental conditions. Wet road surfaces are related only to an increased likelihood of serious casualties in crashes. Compared with summer, autumn and winter are more likely to cause serious casualties but do not significantly affect at-fault behavior. To determine the risk factors and mechanisms for the occurrence of at-fault behaviors and serious casualties in rural areas, we combine the findings of previous studies with the empirical results of this study and conduct discussion.

### 5.1 Driver factors

A comparison of the empirical results of risk factors for causing at-fault crashes and serious casualties in rural areas indicates that, consistent with the conclusions of the previous literature, male drivers are more likely to cause at-fault crashes and serious casualties (8, 17, 19), and the impact of improper passing on serious casualties in crashes is greater in rural areas than in urban areas (21, 56). However, unlike previous literature suggesting that young drivers in rural areas are more likely to be involved in serious road crash injuries (57), our study results show that young drivers aged ≤25 years old are more likely to cause at-fault crashes in rural areas, but the impact on serious casualties is not significant; the better physical fitness of young drivers is considered the reason for the reduced probability of death (18). In addition, we determined the following findings. First, drivers with rural hukou are less likely to cause at-fault crashes in rural areas, but are more likely to cause serious casualties. Secondly, drivers without a valid license are more likely to exhibit at-fault behavior and cause serious casualties in rural areas. Third, migrant workers are more likely than farmers to cause at-fault crashes in rural areas; however, in rural areas, drivers who are workers, migrant workers, and self-employed individuals are less likely than farmer drivers to cause serious casualties.

### 5.2 Vehicle factors

Among all types of vehicles driven in rural areas, truck drivers are more likely than motorcycle drivers to cause at-fault crashes and serious casualties. Therefore, the findings of this study are consistent with those of previous studies (26, 28) regarding truck traffic risk. However, contrary to the view that motorcyclists are more likely to be seriously injured or killed in crashes in rural areas (27), we find that in rural areas, other vehicles, including passenger vehicles, are more likely to cause at-fault crashes than motorcycles. Moreover, vehicles other than passenger vehicles are more likely than motorcycles to cause serious casualties. A possible explanation is the significant improvement in motorcycle traffic safety in China from 2006 to 2014 resulting from the Chinese government’s revision of a series of road safety-related regulations, including two revisions of the Road Traffic Safety Law of People’s Republic of China, three revisions of the Regulations on the Application and Use of Motor Vehicle Driving Licenses, and the two revisions to the Regulations on Road Traffic Safety of Guangdong Province. In addition, our empirical results show the following information. First, vehicles with an unfit safety status are more likely to cause at-fault behavior and serious casualties. Second, vehicles with insurance coverage are much less likely to cause serious casualties.

### 5.3 Road factors

Although the literature indicates that curves and slopes increase the likelihood of traffic crashes in rural areas (33, 34), we do not find a significant impact of curves and slopes on at-fault crashes in rural areas. In addition, curves and slopes reduce the risk of serious casualties in crashes in rural areas, which may be related to the fact that curve crashes are more likely to occur in urban areas during the day (30). In the rural areas of developed countries, there are more crashes at intersections with traffic signals (36). We find that the absence of traffic signals in rural areas in China is more likely to cause at-fault behaviors but less likely to result in serious casualties. A reasonable explanation is that the lack of traffic signals at some intersections in rural China leads to traffic chaos (9), which result in more at-fault behaviors but also make drivers more cautious to avoid serious crashes and casualties. Relatedly, the literature indicates that in rural areas of developed countries, intersections significantly increase the probability and severity of crashes (4, 53). Our empirical results show that drivers in rural areas of China are more likely to cause at-fault crashes on non-intersection roads, and that intersection factors do not have a significant impact on severe casualties. This difference may be related to the fact that Chinese drivers usually exercise more caution when traffic signals are present (55). Consistent with the previous literature, physical road barriers can effectively reduce the occurrence of at-fault behaviors (5, 21). However, physical road barriers also increase the likelihood of causing serious casualties. This may be related to the fact that drivers are not aware of the crash risk with the barriers on rural roads and are still driving at high speeds (58). We find that drivers in rural areas are more likely to cause serious casualties when driving on sand and gravel roads than on asphalt roads, possibly due to difficulties in controlling their vehicles (30). However, our empirical results also show that sand and gravel roads significantly increase the likelihood of drivers causing at-fault crashes in rural areas, and wet road surfaces are more likely to cause serious casualties than dry road surfaces in rural areas, which is different from the findings of previous studies suggesting that drivers are more likely to drive cautiously and reduce the risk of serious casualties under poor pavement conditions (35) and wet road surfaces (25) in rural areas. A possible explanation for the above difference is that drivers who are familiar with rural road traffic conditions tend to accept more risks and engage in more risky behaviors (59, 60).

### 5.4 Environmental factors

We do not find any temporal factor with a significant impact on at-fault behavior in rural areas. However, the empirical results show that the risk of serious casualties during the time periods of 6:00–17:59 and 18:00–23:59 is much lower than that during the time period of 0:00–5:59, which is consistent with the previous literature (31, 61). The empirical results do not confirm an increase in the number of crashes due to winter factors (41, 42), which may be related to the large geographic differences and winter environmental differences, as the relevant literature data come from the state of Washington in the US, which is above 45°N latitude, while the data in this study are from Guangdong Province, China, which is below 24°N latitude. Nevertheless, we reveal that the risk of serious casualties in rural areas in autumn and winter is greater than that in summer,^11^ which may be related to the nationwide agricultural busy season in autumn in China leading to more motor vehicles participating in traffic in rural areas, as well as snowfall amplifying aggressive and risk-taking behaviors (43). The conclusions about the lighting factors are consistent with those of previous studies, indicating that at-fault crashes and serious casualties are more likely to occur at nighttime without street lighting (62–64). However, regarding the negative impact of adverse weather conditions on traffic safety (24, 44), the empirical results of this study show that adverse weather conditions do not significantly affect at-fault crash behavior; furthermore, compared to good weather conditions, adverse weather conditions are less likely to cause serious casualties, but poor visibility significantly increases the likelihood of drivers causing at-fault crashes in rural areas. Regarding terrain factors, we find that mountainous terrain more likely leads drivers to cause at-fault crashes and serious casualties in rural areas, which is in agreement with the review of previous literature that mountainous roads are unfavorable for safe driving and result in more dangerous driving behavior (5).

Additionally, we find that single-vehicle crashes have a greater impact on the serious crash casualties in rural China than multivehicle crashes, which is consistent with the conclusion of Wang et al. (18). In contrast to the findings in the literature that the impact of fatigue driving on serious casualties in rural areas is much greater than that in urban areas (20, 63), we find that fatigue driving has no significant impact in rural areas but has a significant impact in urban areas. A reasonable explanation is that truck drivers are a high-risk group for fatigue driving behavior (48). According to our data, truck drivers account for 18.4% of all drivers in rural areas and 26.2% in urban areas. Therefore, the impact of fatigue driving in urban areas is greater than that in rural areas. The empirical results show that speeding and drunk driving have significant impacts on serious casualties in crashes in both rural areas and urban areas, which may be related to the annual increase in per capita motor vehicle ownership in rural and urban areas (65).

### 5.5 Comparison of risk factors in rural and urban areas

We compare the risk factors between rural areas and urban areas in terms of at-fault crash behavior and serious casualties, and the results show that factors such as gender, hukou, driver’s license, vehicle safety status, vehicle type, and traffic signal conditions are the most significant. Notably, male gender, not having a driver’s license, unsafe vehicles, and trucks are the top four risk factors because they significantly increase the likelihood of causing at-fault crash behaviors and serious casualties in both rural and urban areas. In both rural and urban areas, drivers with rural hukou are less likely to cause at-fault crashes but more likely to cause serious casualties. However, the absence of traffic signals makes drivers more likely to cause at-fault crashes, but these are less likely to result in serious casualties in crashes.

The impacts of some risk factors in rural and urban areas are the same. There are five common factors that are more likely to cause at-fault crashes: drivers aged ≤25 years old, migrant worker drivers, passenger vehicles, cement pavement, and poor visibility. There are two common factors that are less likely to cause at-fault behaviors: intersections and physical road barriers. There are seven common factors that are more likely to cause serious casualties: wet road surfaces, mountainous terrain, nighttime without street lighting, autumn, winter, speeding, and drunk driving. There are six common factors that are less likely to cause serious casualties: vehicles with insurance, curves and slopes, hilly terrain, all time periods except 0:00–5:59, adverse weather conditions, and multivehicle crashes.

A key finding is that factors such as driver occupation, road surface, and collision type exert opposite effects on severe casualties in rural versus urban areas. Compared to farmer drivers, migrant workers and self-employed drivers are less likely to cause severe casualties in rural areas but more likely to do so in urban areas. Compared to rural areas, urban areas feature complex road network structures, high traffic volumes, and higher speed limits. Migrant workers and self-employed drivers in urban areas are more likely to face strong time constraints and unfamiliar environments, increasing the likelihood of severe injury or fatality accidents. Gravel roads and single-vehicle collisions increase the probability of severe injury or fatality in rural areas but reduce it in urban areas. Gravel roads predominantly exist in rural areas and are less common in urban areas. Consequently, urban drivers encountering unfamiliar road surfaces are more likely to engage in compensatory driving behaviors. Furthermore, gravel roads in urban areas are more frequently located in low-traffic density zones, making them less likely to cause severe casualties. Rural areas commonly lack adequate traffic safety infrastructure, feature vehicles with lower safety specifications, and suffer from insufficient emergency response and medical treatment capabilities. These factors make drivers more vulnerable to fatal injuries in single-vehicle accidents and hinder timely access to professional medical care. Additionally, lower traffic volumes in rural areas make accidents harder to detect promptly, potentially triggering secondary collisions.

In addition, vehicles without license plates are less likely to cause at-fault crashes and serious casualties in urban areas, but their impact in rural areas is insignificant in the two models; therefore, license plates are a unique risk factor in urban areas. Finally, our study shows that the influences of street lighting at night and spring factors are not significant in rural and urban areas.

### 5.6 Differences in traffic risks for rural areas between developing and developed countries

First, in previous studies of the rural areas in developed countries, factors of sand and gravel pavement and wet road surface are considered to encourage cautious driving and thus reduce traffic risks. However, in the rural areas of China, these two factors are more likely to cause serious casualties; furthermore, sand and gravel pavement in rural China makes drivers more likely to cause at-fault crashes rather than driving carefully. Previous literature suggests that drivers are more likely to drive cautiously on poor road pavement (35) and wet road surfaces (25) in rural areas, resulting in a lower probability of serious injuries on rural roads than on asphalt roads (24) and dry road conditions (27). However, drivers in rural China do not demonstrate cautious behavior under poor road pavement and surface conditions; compared with asphalt pavement, sand and gravel pavement significantly increases the likelihood of causing at-fault crashes and serious casualties for drivers in rural areas, and wet road surfaces are more likely than dry road surfaces to cause serious casualties.

Second, regarding the risk factors that are unfavorable for traffic in rural areas of developed countries, previous studies have suggested that young drivers, motorcycles, and fatigue driving are associated with serious casualties; that traffic signals, weekdays, and adverse weather are associated with more traffic crashes; and that road alignment and intersections are associated with both nighttime at-fault crashes and serious casualties. However, the impact of these factors is not significant in rural China. In terms of driver factors, previous literature has shown that young drivers in developed countries are more likely to be involved in serious road crash injuries in rural areas (18), and that fatigue driving is more common (63) and dangerous (20) in rural areas than in urban areas. However, we find that young drivers aged ≤25 years old in rural China are not a significant factor in causing serious casualties in rural areas, and fatigue driving is not a significant factor influencing serious casualties in crashes in rural areas but significantly affects only those in urban areas. In terms of vehicle factors, motorcycles in developed countries increase the risk of serious casualties in rural areas (27, 29). However, among all vehicles involved in crashes in rural China, motorcycles have the lowest likelihood of causing at-fault crashes and pose a smaller risk of serious casualties compared to all other vehicles except passenger vehicles. In terms of road factors, previous studies have shown that curves, slopes (9, 33), and intersections (4, 25) are more likely to cause crashes and serious casualties, and that traffic signals are more likely to distract rural drivers (5) and cause crashes (36). However, we find that factors of non-straight roads, traffic signals, and intersections do not increase the risk of at-fault behaviors or serious casualties in rural China, and that non-straight roads instead reduce the risk of serious casualties in crashes. In terms of environmental factors, previous studies have shown that in developed countries, weekdays and adverse weather conditions are more likely to lead to crashes (24, 44). However, our empirical results show that no time factor significantly affects at-fault behaviors in rural China, that adverse weather conditions do not increase at-fault behaviors in rural areas, and that the risk of serious casualties is instead lower than that under good weather conditions.

Third, previous studies have paid little attention to hukou, driver license, occupation, vehicle safety status, and insurance. This may be due to a lack of relevant data or the absence of hukou systems in some developed countries. This study reveals that these factors all lead to significant differences in the rural areas of developing countries. First, we find that drivers without valid licenses and unsafe vehicles significantly increase the likelihood of at-fault crashes and serious casualties in rural areas. Therefore, the supervision is urgently needed for drivers and vehicles operating illegally in the rural areas of developing countries. Second, our empirical results indicate that to improve traffic safety in rural areas, the issue of drivers with rural hukou and occupations as farmers, who are more likely to cause crashes and serious casualties, should be addressed. This is particularly urgent for developing countries like China with a large rural population, of whom a significant proportion are engaged in farming. Lastly but equally important, migrant workers are more likely than farmers to cause at-fault crashes, and vehicles with insurance are less likely to cause serious casualties than vehicles without insurance. This may suggest that at least nearly one-fifth of drivers and nearly one-third of vehicles in rural China may already pose traffic safety risks before they hit the road.

## 6 Policy recommendations and research prospects

The current global reduction in road fatalities is far from reaching the target set by the Global Plan for the Decade of Action for Road Safety (14). Therefore, there should be a focus on road safety in low- and middle-income countries (66). Based on the research on the risk factors for at-fault behaviors and the severity of casualties in rural China and a comparison study with urban areas, effective and feasible policy recommendations are proposed in accordance with the characteristics of the risk factors for drivers, vehicles, roads, and the environment in rural areas, with the aim of reducing the likelihood of crashes and serious casualties in rural areas of developing countries.

First, male drivers and drivers without licenses in rural China are more likely to exhibit at-fault behavior and cause serious casualties; speeding is very likely to cause serious casualties; and factors such as age ≤25 years, rural hukou, and occupation as migrant workers have significant impacts. Therefore, the establishment of a comprehensive supervision system for driver license operations is urgently needed, as is a strict crack down on illegal driving behavior. Additionally, the safety awareness of drivers should be enhanced in a targeted manner. The traffic management department should strengthen the supervision and management of driver tests and the issuance and use of driving licenses in rural areas to compensate for the insufficient road traffic supervision in rural areas (67, 68). In particular, the inspection of improper driving behaviors such as speeding, drunk driving, and improper overtaking should be strengthened, and the relevant prohibitions on drivers with illegal driving behaviors should be strictly implemented to prevent these drivers from applying for driving licenses. Considering that male drivers account for 92.6% of the total number of drivers and that male drivers and drivers without licenses are important risk factors that increase the likelihood of at-fault crashes and serious casualties in rural and urban areas, special efforts should be made to strengthen supervision and inspections of male drivers’ license application and usage as well as traffic violations in rural areas. Since the factors of rural hukou and farmers’ occupation are more likely to account for serious casualties, extra attention should be given to the fact that farmers living in rural areas are likely to account for serious crash casualties in rural areas due to their familiarity with rural road traffic and more risky behaviors (59, 60); thus, safety awareness should be strengthened for local drivers. In addition, training and other means should be used to reduce the crash behavior of young drivers aged ≤25 years old and migrant workers.

Second, unsafe vehicles have a significant impact on the increase in the likelihood of at-fault crashes and serious casualties in rural China. Although the number of motorcycles owned by rural residents reaches as high as 49.9 per 100 households (65), the likelihood of at-fault crashes and serious casualties due to truck factors in rural China is much greater than that of motorcycles. Therefore, the vehicle safety qualifications in rural areas should be strictly examined, and traffic risks posed by trucks in rural areas should be given attention. Vehicle safety has a significant impact on the severity of road traffic crash casualties (69). We find that unsafe vehicles have a significant impact on the increase in the likelihood of crashes and serious casualties in rural areas. Therefore, traffic management departments should strengthen the inspection of vehicle safety performance in rural areas and remove illegally assembled vehicles and scrap vehicles that have reached the scrapping standard. Vehicles that do not provide vehicle insurance certificates as required should not pass inspection. Particularly, vehicles exceeding their service life, failing to meet safety technical standards, and failing to obtain inspection qualification marks within the prescribed period should be compulsorily scrapped. Since trucks significantly increase the likelihood of at-fault traffic crashes and serious casualties in crashes in rural areas, attention should be focused on the issues of truck overloading, illegal production, and modification in rural areas. Furthermore, efforts should be made to establish detection lanes and traffic signs to strengthen the supervision of overloaded trucks and to include serious violations of truck overloading into criminal law regulations to enhance the deterrent effect on truck drivers in rural areas.

Third, special attention should be paid to the issue of sand and gravel pavement, which significantly increases the likelihood of at-fault crashes and serious casualties in rural areas of China. Attention should also be given to traffic signals and cement pavement to reduce at-fault behaviors, as well as to the physical road barriers and wet road surfaces to reduce serious casualties. Therefore, pavement quality should be improved, and road traffic safety mechanisms should be optimized to enhance driver awareness of road risk in rural areas. The difference in road risk factors between urban and rural areas deserves special attention. In rural areas, sand and gravel pavement is more likely to cause single-vehicle crashes (64) and at-fault behaviors, and both sand and gravel pavement and single-vehicle crashes increase the likelihood of serious casualties in rural areas. However, neither is likely to cause serious casualties in urban areas. Therefore, regarding roads in rural areas, priority should be given to paving roads and establishing long-term mechanisms for their management and maintenance. Compared with developed countries, developing countries should enhance the risk awareness and emphasis of drivers in rural areas on poor pavement and road surface conditions to reduce the likelihood of at-fault behaviors caused by cement pavement and serious casualties caused by wet surfaces. In addition, considering the general lack of road barriers in rural areas (21), safety education regarding road barriers and road signage should be strengthened to reduce the likelihood of serious casualties. Given that the absence of traffic signals leads to an increase in at-fault behaviors in rural areas, the updating and improvement of traffic signs may have positive impacts on road safety (70); in particular, it is important to promptly improve and correct defects such as missing traffic signals and erroneous signage (71).

Fourth, considering the significant impact of mountainous terrain and nighttime without street lighting in rural areas of China on both at-fault crash behavior and severe casualties, as well as the increased likelihood of at-fault crash behavior and severe casualties in rural areas due to visibility and seasonal factors, respectively, the traffic infrastructure construction in rural areas should be improved, and special traffic supervision measures should be implemented under specific conditions. Unfavorable terrain and lighting conditions have a significant impact on the increase in likelihood of at-fault crash behaviors and serious casualties in rural areas. Therefore, continuous efforts should be made to construct and improve safety protection mechanisms and monitoring equipment on mountainous roads and to accelerate the construction of traffic infrastructure such as the rural road lighting projects (12). Traffic management departments should strengthen traffic supervision in rural areas with poor visibility and during the time period of 0:00 to 5:59 daily and during the autumn and winter seasons. Considering the issues of insufficient traffic police supervision and speed camera enforcement in rural areas (5, 34), as well as the low traffic volume in rural areas (9, 63), measures such as “equipment-based fixed-point monitoring combined with dynamic traffic police patrols for prevention and control” may effectively improve traffic conditions in rural areas.

Fifth, this study offers new perspectives for rural road safety policies in China, including expanding driving schools, implementing differentiated training, addressing gravel roads, and preventing bicycle accidents. First, rural areas in China and other developing countries face issues such as insufficient driving schools, uneven spatial distribution, and scarce training resources. This results in rural drivers lacking essential driving skills and even operating vehicles without licenses. This study proposes differentiated approaches to driver training in rural areas, such as requiring migrant workers to undergo urban–rural road difference training and incorporating content on rural road specificities into training for rural residents. Second, current policies underestimate the impact of gravel roads on traffic accidents. For instance, the 2025 Rural Highway Regulations^12^ primarily address safety hazards in sharp curves, steep slopes, waterfronts, cliff edges, intersections, bridges, tunnels, and sections passing through villages and towns—but exclude gravel surfaces. Finally, existing policies primarily focus on preventing multi-vehicle accident risks but fail to recognize the link between rural roads and high fatality rates in single-vehicle accidents. There is an urgent need to conduct safety risk assessments of existing rural roads based on the Standards for Rural Highway Condition Assessment.^13^ Priority should be given to addressing risk hazards on high-risk sections, implementing highway safety life protection projects, and establishing rapid response and rescue networks for traffic accidents.

The data in this study are from the Road Traffic Crash Database of the Ministry of Public Security specifically, the Case Reports on Specific Events by Traffic Management Departments. The data of Guangdong Province, which ranks second in both the number of traffic crashes and the number of deaths nationwide, are selected to ensure the representativeness of the data, comparability of urban–rural differences, accuracy of conclusions, and effectiveness of recommendations. However, this study is not without limitations. First, due to the limited entries of information in the database and issue of some missing data, we are unable to analyze the differences in risk factors between two-lane and multi-lane traffic in rural areas (5, 35), as well as the impacts of presence and width of road shoulders (33, 72) and speed limits on rural roads (28, 73) on traffic safety in rural areas. In addition, differences in drivers’ behavior and attitudes are considered important influencing factors (49). Therefore, further research is needed to explore the differential influences of occupational differences in rural areas on traffic risky behavior and attitudes to analyze how the risk awareness, risk perception, and risk propensity of specific occupational groups of drivers in rural areas are associated with risk factors for at-fault crash behaviors and serious casualties.

## Data Availability

The raw data supporting the conclusions of this article will be made available by the authors, without undue reservation.

## Footnotes

1 Xinhua News Agency: “Xi Jinping: Building Prosperous Roads, Fulfilling the Dream of Common Prosperity,” China Government Website, April 28, 2014, https://www.gov.cn/govweb/xinwen/2014-04/28/content_2668189.htm.

2 Alexander Ayertey Odonkor: High-quality transport infrastructure strengthens China’s rural economies, China Daily, 2022-11-15, https://global.chinadaily.com.cn/a/202211/15/WS637331c5a310491754329ce3.html.

3 China News Network: “Focusing on Road Traffic Safety: Over 40% of Rural Roads Have Safety Hazards,” December 22, 2016, https://www.chinanews.com.cn/sh/2016/12-22/8101093.shtml.

4 This database is the only official and legal source of traffic data in China. It is operated in accordance with the Regulations on the Use and Management of the National Road Traffic Crash Processing Information System. Within 24 h of completing on-site investigation of road traffic crashes, traffic police input relevant information into the National Road Traffic Crash Processing Information System according to the Road Traffic Crash Information Codes developed by the Computer and Information Processing Standardization Technical Committee of the Ministry of Public Security. The database includes detailed information on each crash, such as characteristics of the parties involved, extent of casualties, vehicle characteristics, road conditions, time and environment of the crash, and other specific indicators. These indicators cover various aspects including the severity of traffic crash, crash forms, causes of crashes, extent of motor vehicle damage, types of responsibilities of the parties involved, severity of injuries to the parties involved, parts of the body injured, purposes of travel, status of motor vehicle operation, types of motor vehicle drivers, and insurance status of the parties involved (48, 55, 74).

5 China National Radio: “The ‘Hundreds, Thousands, and Millions Project’: Guangdong’s Approach to Addressing Imbalances in Urban–Rural and Regional Development,” December 23, 2025, https://news.cnr.cn/native/gd/20251223/t20251223_527469789.shtml.

6 To avoid interference from variable definitions and model selection on the results, we defined the outcome variable as an ordinal variable in Appendix Table A1 (where minor injuries and no injuries served as the control group, assigned a value of 0; severe injuries were assigned a value of 1; and death was assigned a value of 2). We then conducted a robustness test using an ordered logit model, and the results were consistent with our baseline regression.

7 Wenqin Li: “Artificial Fog Dissipation and Its Operation Method,” China Meteorological Administration website, November 5, 2020, https://www.cma.gov.cn/2011xzt/2012zhuant/20120412/2012041211/201204/t20120417_3093323.html.

8 Chinese Government Website: Regulations for the Implementation of the Road Traffic Safety Law of the People’s Republic of China, https://www.gov.cn/gongbao/content/2019/content_5468932.htm.

9 It should be pointed out that while random-effects Logit, multilevel Logit, or mixed-effects models do not theoretically require panel data, their parameter heterogeneity identification relies primarily on distributional assumptions in the absence of repeated observations on the same driver, vehicle, or road unit. Consequently, the interpretation of results becomes highly sensitive to model specification. Therefore, in studies aiming for average-effects identification with clear policy implications, employing the standard Logit model represents a more transparent and robust approach.

10 We further validate our model selection through multiple robustness checks in the Appendix, including estimating an ordered logit model (Appendix Table A1) and incorporating city-level fixed effects with clustered standard errors at the city level (Appendix Table A2). Notably, the inclusion of city-level fixed effects substantially controls for unobservable regional-level confounders that do not vary over time. The corresponding results indicate that the main conclusions of this paper are not dependent on specific model specifications.

11 China Government Website: “Busy Farming in the Golden Autumn Season,” October 9, 2023, https://www.gov.cn/yaowen/tupian/202310/content_6907909.htm#1.

12 China Government Website: Rural Roads Regulations, July 16, 2025, https://www.gov.cn/zhengce/content/202507/content_7033173.htm.

13 China Government Website: Standards for Rural Highway Condition Assessment, May 20, 2024, https://www.gov.cn/zhengce/zhengceku/202406/content_6956101.htm.
