# Supplementary material for: Risk factors for traffic violations and serious crash casualties in rural areas: a comparison of urban–rural differences in China
Source: Front Public Health. 2026 Jan 29;14:1754725. doi: 10.3389/fpubh.2026.1754725 (PMC12894276; doi:10.3389/fpubh.2026.1754725)
Supplement: Supplementary file 1 [file Data_Sheet_1.docx]

**Appendix**

Table A1. Results of the ordered logit model.

|  |  | (1) | | (2) | |
| --- | --- | --- | --- | --- | --- |
|  |  | Rural areas | | Urban areas | |
| *Human factors* |  |  | |  | |
| Gender (base: female) |  | 1.346*** | | 1.382*** | |
|  |  | (0.059) | | (0.049) | |
| Age (base: > 25) |  | 0.956* | | 0.942*** | |
|  |  | (0.024) | | (0.019) | |
| *Hukou* origin (base: urban) |  | 1.210*** | | 1.244*** | |
|  |  | (0.028) | | (0.023) | |
| State of driving license (base: valid license) |  | 1.199*** | | 1.236*** | |
|  |  | (0.036) | | (0.030) | |
| Occupation (base: farmers) |  |  | |  | |
| General staffs |  | 1.050 | | 1.034 | |
|  |  | (0.058) | | (0.038) | |
| Workers |  | 0.752*** | | 1.008 | |
|  |  | (0.026) | | (0.026) | |
| Migrant workers |  | 0.828*** | | 1.117*** | |
|  |  | (0.028) | | (0.033) | |
| Self-employed |  | 0.895*** | | 1.139*** | |
|  |  | (0.037) | | (0.032) | |
| Others |  | 0.966 | | 1.056** | |
|  |  | (0.028) | | (0.024) | |
| *Vehicle factors* |  | |  | |  |
| Vehicle safety status (base: fit) |  | 1.432*** | | 1.503*** | |
|  |  | (0.049) | | (0.041) | |
| License plate status (base: with a license plate) |  | 1.057* | | 0.936** | |
|  |  | (0.032) | | (0.024) | |
| Insurance (base: invalid) |  | 0.816*** | | 0.816*** | |
|  |  | (0.020) | | (0.017) | |
| Vehicle type (base: motorcycle) |  |  | |  | |
| Passenger vehicle |  | 1.049 | | 1.132*** | |
|  |  | (0.033) | | (0.026) | |
| Truck |  | 1.965*** | | 1.705*** | |
|  |  | (0.062) | | (0.039) | |
| Others |  | 2.008*** | | 2.023*** | |
|  |  | (0.096) | | (0.069) | |
| *Road factors* |  | |  | |  |
| Intersection (base: no) |  | 0.991 | | 0.870*** | |
|  |  | (0.026) | | (0.018) | |
| Road alignment (base: smooth) |  | 0.750*** | | 0.732*** | |
|  |  | (0.020) | | (0.014) | |
| Traffic signal conditions (base: having traffic signal) |  | 0.811*** | | 0.733*** | |
|  |  | (0.019) | | (0.017) | |
| Whether there are physical barriers in roads (base: no) |  | 1.243*** | | 1.016 | |
|  |  | (0.030) | | (0.017) | |
| Road surface (base: dry) |  |  | |  | |
| Wet |  | 1.132*** | | 1.242*** | |
|  |  | (0.046) | | (0.035) | |
| Others |  | 1.381*** | | 1.036 | |
|  |  | (0.094) | | (0.056) | |
| Road structure (base: pitch) |  |  | |  | |
| Cement |  | 1.027 | | 0.948*** | |
|  |  | (0.027) | | (0.016) | |
| Gravel |  | 1.431*** | | 0.550** | |
|  |  | (0.145) | | (0.148) | |
| Others |  | 0.739*** | | 0.622*** | |
|  |  | (0.078) | | (0.052) | |
| *Environmental factors* |  |  | |  | |
| Terrain (base: plain) |  |  | |  | |
| Hill |  | 0.930*** | | 0.949*** | |
|  |  | (0.023) | | (0.016) | |
| Mountain |  | 1.409*** | | 1.318*** | |
|  |  | (0.050) | | (0.030) | |
| Street-light condition (base: daylight) |  |  | |  | |
| Dark but lighted |  | 0.978 | | 1.093 | |
|  |  | (0.093) | | (0.073) | |
| Dark |  | 1.486*** | | 1.852*** | |
|  |  | (0.141) | | (0.124) | |
| Weather condition (base: good) |  | 0.914*** | | 0.889*** | |
|  |  | (0.026) | | (0.019) | |
| Visibility level (base: good) |  | 1.003 | | 0.892*** | |
|  |  | (0.022) | | (0.014) | |
| Weekends (base: weekdays) |  | 1.017 | | 1.052*** | |
|  |  | (0.022) | | (0.017) | |
| Time of day (base: 0:00-5:59) |  |  | |  | |
| 6:00-17:59 |  | 0.542*** | | 0.673*** | |
|  |  | (0.053) | | (0.045) | |
| 18:00-23:59 |  | 0.553*** | | 0.641*** | |
|  |  | (0.020) | | (0.015) | |
| Season (base: summer) |  |  | |  | |
| Spring |  | 1.019 | | 0.999 | |
|  |  | (0.028) | | (0.020) | |
| Autumn |  | 1.101*** | | 1.105*** | |
|  |  | (0.032) | | (0.024) | |
| Winter |  | 1.113*** | | 1.133*** | |
|  |  | (0.030) | | (0.022) | |
| *Crash condition* |  |  | |  | |
| Crash type (base: others) |  |  | |  | |
| Single-vehicle collision |  | 1.830*** | | 0.945 | |
|  |  | (0.085) | | (0.036) | |
| Multiple-vehicle collision |  | 0.869*** | | 0.691*** | |
|  |  | (0.025) | | (0.016) | |
| Traffic violation (base: others) |  |  | |  | |
| Speeding |  | 2.414*** | | 1.731*** | |
|  |  | (0.147) | | (0.070) | |
| Drunk driving |  | 1.456*** | | 1.720*** | |
|  |  | (0.103) | | (0.105) | |
| Improper overtaking |  | 1.138** | | 1.059 | |
|  |  | (0.070) | | (0.046) | |
| Fatigue driving |  | 1.263 | | 1.458*** | |
|  |  | (0.197) | | (0.116) | |
| Pseudo R-squared |  | 0.045 | | 0.043 | |
| *N* |  | 58987 | | 92102 | |

Notes: Exponentiated coefficients; Standard errors in parentheses. Base indicates the reference group.

^*^ *p* < 0.1, ^**^ *p* < 0.05, ^***^ *p* < 0.01


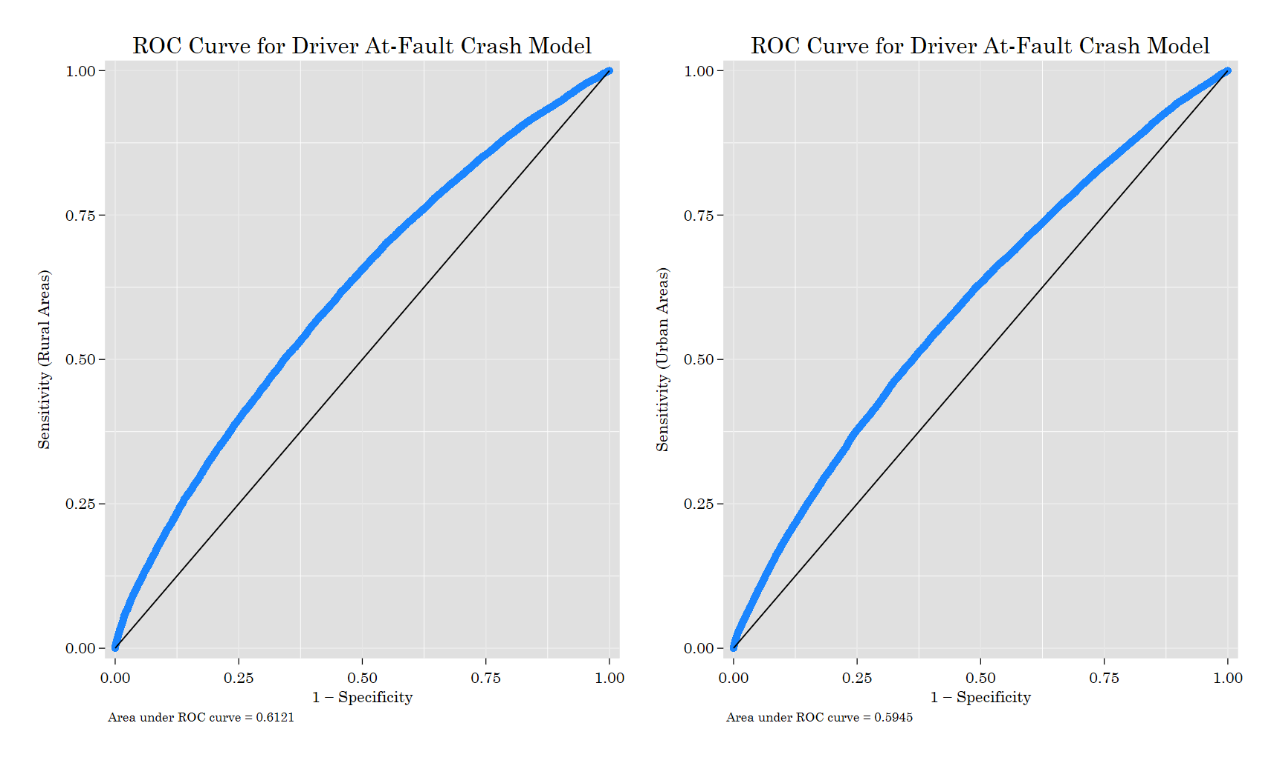


Figure A1. ROC Curve for Driver At-Fault Crash Model


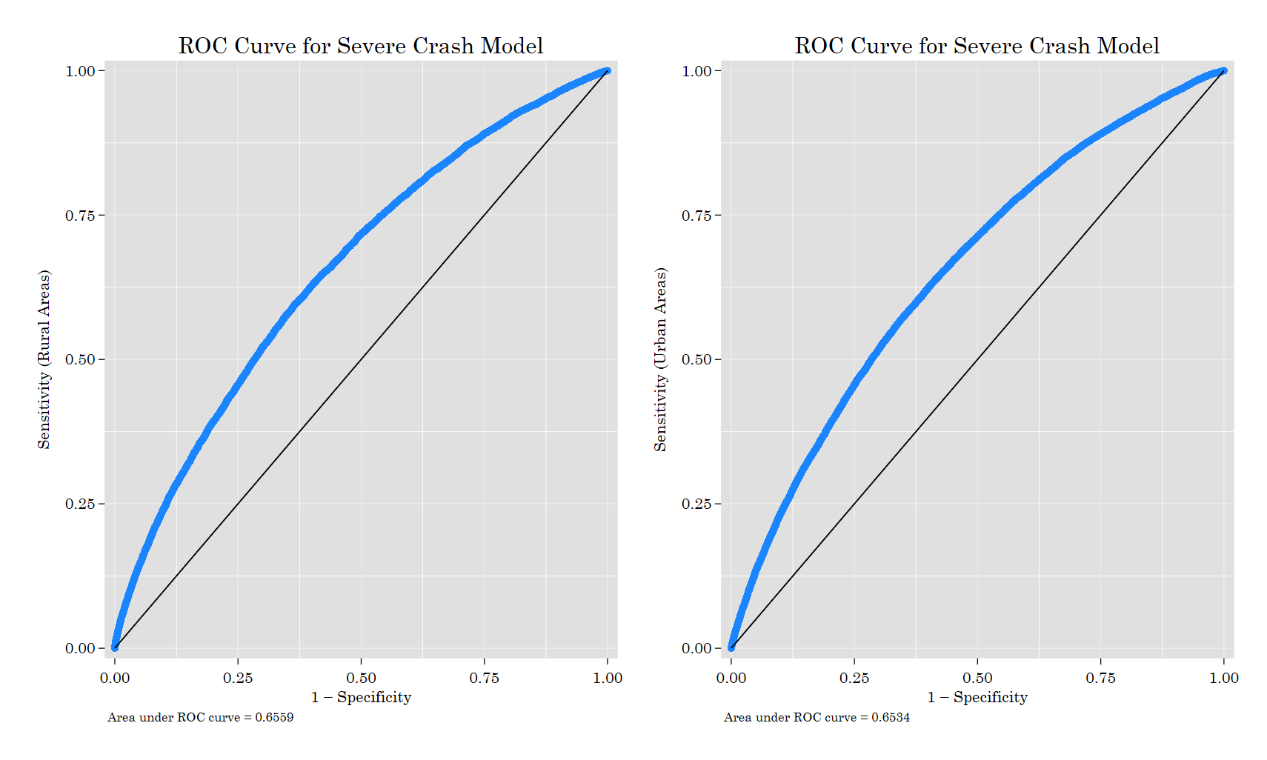


Figure A2. ROC Curve for Severe Crash Model

Table A2. Fixed-effects estimates with clustered standard errors.

|  | (1) | (2) |  | (3) | (4) |
| --- | --- | --- | --- | --- | --- |
|  | Driver at-fault crash model | |  | Severe crash model | |
|  | Rural areas | Urban areas |  | Rural areas | Urban areas |
| *Human factors* |  |  |  |  |  |
| Gender (base: female) | 1.221*** | 1.221*** |  | 1.349*** | 1.382*** |
|  | (0.042) | (0.037) |  | (0.086) | (0.050) |
| Age (base: > 25) | 1.288*** | 1.273*** |  | 0.966 | 0.962* |
|  | (0.041) | (0.027) |  | (0.024) | (0.020) |
| *Hukou* origin (base: urban) | 0.771*** | 0.808*** |  | 1.149*** | 1.194** |
|  | (0.031) | (0.014) |  | (0.062) | (0.095) |
| State of driving license (base: valid license) | 1.457*** | 1.383*** |  | 1.180*** | 1.226*** |
|  | (0.113) | (0.097) |  | (0.045) | (0.046) |
| Occupation (base: farmers) |  |  |  |  |  |
| General staffs | 0.938 | 0.938* |  | 1.082 | 1.042 |
|  | (0.045) | (0.031) |  | (0.106) | (0.085) |
| Workers | 0.941 | 0.941 |  | 0.785*** | 0.985 |
|  | (0.057) | (0.040) |  | (0.070) | (0.074) |
| Migrant workers | 1.105*** | 1.197*** |  | 0.863 | 1.086 |
|  | (0.031) | (0.046) |  | (0.154) | (0.222) |
| Self-employed | 0.941* | 0.870*** |  | 0.952 | 1.169 |
|  | (0.033) | (0.040) |  | (0.144) | (0.119) |
| Others | 0.985 | 0.934** |  | 0.968 | 1.039 |
|  | (0.024) | (0.028) |  | (0.128) | (0.121) |
| *Vehicle factors* |  |  |  |  |  |
| Vehicle safety status (base: fit) | 1.136* | 1.395*** |  | 1.358** | 1.351*** |
|  | (0.084) | (0.097) |  | (0.166) | (0.069) |
| License plate status (base: with a license plate) | 1.025 | 0.980 |  | 1.015 | 0.942 |
|  | (0.057) | (0.067) |  | (0.059) | (0.039) |
| Insurance (base: invalid) | 1.022 | 1.047* |  | 0.860** | 0.846** |
|  | (0.041) | (0.026) |  | (0.062) | (0.064) |
| Vehicle type (base: motorcycle) |  |  |  |  |  |
| Passenger vehicle | 2.231*** | 2.015*** |  | 0.973 | 1.060 |
|  | (0.356) | (0.239) |  | (0.070) | (0.052) |
| Truck | 2.117*** | 1.814*** |  | 1.755*** | 1.598*** |
|  | (0.296) | (0.202) |  | (0.129) | (0.078) |
| Others | 1.544*** | 1.204* |  | 1.778*** | 1.923*** |
|  | (0.118) | (0.128) |  | (0.122) | (0.162) |
| *Road factors* |  |  |  |  |  |
| Intersection (base: no) | 0.933** | 0.956* |  | 1.017 | 0.925 |
|  | (0.030) | (0.025) |  | (0.103) | (0.067) |
| Road alignment (base: smooth) | 1.021 | 1.056** |  | 0.851* | 0.870** |
|  | (0.034) | (0.023) |  | (0.071) | (0.056) |
| Traffic signal conditions (base: having traffic signal) | 1.193*** | 1.158*** |  | 0.770*** | 0.726*** |
|  | (0.020) | (0.033) |  | (0.075) | (0.074) |
| Whether there are physical barriers in roads (base: no) | 0.892*** | 0.888*** |  | 1.197*** | 1.025 |
|  | (0.028) | (0.020) |  | (0.066) | (0.066) |
| Road surface (base: dry) |  |  |  |  |  |
| Wet | 0.961 | 0.953** |  | 1.005 | 1.187*** |
|  | (0.037) | (0.020) |  | (0.051) | (0.064) |
| Others | 1.068 | 1.045 |  | 1.379*** | 1.132 |
|  | (0.068) | (0.038) |  | (0.127) | (0.186) |
| Road structure (base: pitch) |  |  |  |  |  |
| Cement | 1.144*** | 1.213*** |  | 0.968 | 0.938 |
|  | (0.046) | (0.051) |  | (0.046) | (0.085) |
| Gravel | 1.456*** | 1.206 |  | 1.310 | 0.624 |
|  | (0.206) | (0.184) |  | (0.224) | (0.198) |
| Others | 1.371** | 1.103 |  | 0.704* | 0.745 |
|  | (0.174) | (0.067) |  | (0.148) | (0.412) |
| *Environmental factors* |  |  |  |  |  |
| Terrain (base: plain) |  |  |  |  |  |
| Hill | 0.991 | 0.995 |  | 1.005 | 1.024 |
|  | (0.041) | (0.044) |  | (0.176) | (0.143) |
| Mountain | 1.038 | 1.013 |  | 1.335* | 1.278** |
|  | (0.058) | (0.044) |  | (0.225) | (0.147) |
| Street-light condition (base: daylight) |  |  |  |  |  |
| Dark but lighted | 1.026 | 1.049 |  | 1.071 | 1.134 |
|  | (0.088) | (0.045) |  | (0.163) | (0.160) |
| Dark | 1.178* | 0.958 |  | 1.541*** | 1.792*** |
|  | (0.099) | (0.041) |  | (0.198) | (0.288) |
| Weather condition (base: good) | 1.013 | 0.969 |  | 1.020 | 0.921** |
|  | (0.021) | (0.022) |  | (0.047) | (0.035) |
| Visibility level (base: good) | 1.036 | 1.035 |  | 1.006 | 0.880*** |
|  | (0.027) | (0.031) |  | (0.078) | (0.040) |
| Weekends (base: weekdays) | 1.000 | 0.984 |  | 1.024 | 1.047* |
|  | (0.013) | (0.012) |  | (0.022) | (0.029) |
| Time of day (base: 0:00-5:59) |  |  |  |  |  |
| 6:00-17:59 | 0.954 | 1.029 |  | 0.646*** | 0.732** |
|  | (0.094) | (0.062) |  | (0.088) | (0.099) |
| 18:00-23:59 | 1.062 | 1.146*** |  | 0.611*** | 0.689*** |
|  | (0.079) | (0.060) |  | (0.034) | (0.030) |
| Season (base: summer) |  |  |  |  |  |
| Spring | 0.980 | 1.000 |  | 1.021 | 0.984 |
|  | (0.020) | (0.014) |  | (0.034) | (0.024) |
| Autumn | 1.019 | 1.037*** |  | 1.070* | 1.090** |
|  | (0.021) | (0.011) |  | (0.042) | (0.045) |
| Winter | 0.980 | 1.013 |  | 1.110** | 1.128*** |
|  | (0.019) | (0.019) |  | (0.058) | (0.035) |
| *Crash condition* |  |  |  |  |  |
| Crash type (base: others) |  |  |  |  |  |
| Single-vehicle collision |  |  |  | 1.679*** | 1.014 |
|  |  |  |  | (0.140) | (0.125) |
| Multiple-vehicle collision |  |  |  | 0.886** | 0.741*** |
|  |  |  |  | (0.047) | (0.045) |
| Traffic violation (base: others) |  |  |  |  |  |
| Speeding |  |  |  | 2.450*** | 1.774*** |
|  |  |  |  | (0.321) | (0.169) |
| Drunk driving |  |  |  | 1.432 | 1.682** |
|  |  |  |  | (0.376) | (0.421) |
| Improper overtaking |  |  |  | 1.186 | 1.048 |
|  |  |  |  | (0.147) | (0.102) |
| Fatigue driving |  |  |  | 1.237 | 1.394** |
|  |  |  |  | (0.318) | (0.235) |
| Pseudo R-squared | 0.030 | 0.022 |  | 0.072 | 0.070 |
| AUC | 0.618 | 0.599 |  | 0.686 | 0.681 |
| *N* | 58987 | 92102 |  | 58987 | 92102 |

Notes: Exponentiated coefficients; Standard errors in parentheses. Base indicates the reference group.

^*^ *p* < 0.1, ^**^ *p* < 0.05, ^***^ *p* < 0.01
